# Ethnobotany of the Himalayas: Safeguarding Medical Practices and Traditional Uses of Kashmir Regions

**DOI:** 10.3390/biology10090851

**Published:** 2021-08-31

**Authors:** Mudasir Nazir Bhat, Bikarma Singh, Opender Surmal, Bishander Singh, Vijay Shivgotra, Carmelo Maria Musarella

**Affiliations:** 1 Academy of Scientific and Innovative Research (AcSIR), Ghaziabad 201002, Uttar Pradesh, India; mudii644@gmail.com (M.N.B.); rajputop225@gmail.com (O.S.); 2 Plant Sciences (Biodiversity and Applied Botany Division), CSIR-Indian Institute of Integrative Medicine, Jammu 180001, Jammu and Kashmir, India; 3 Botanic Garden Division, CSIR-National Botanical Research Institute (NBRI), Rana Pratap Marg, Lucknow 226001, Uttar Pradesh, India; 4 Department of Botany, Veer Kunwar Singh University, Ara 802301, Bihar, India; bishander85@gmail.com; 5 Department of Biostatistics, University of Jammu, Baba Saheb Ambedkar Road, Jammu 180006, Jammu and Kashmir, India; vijayshivgotra@gmail.com; 6 Department of Agraria, Mediterranea University of Reggio Calabria, Feo di Vito Snc, 89122 Reggio Calabria, Italy; carmelo.musarella@unirc.it

**Keywords:** ethnomedicinal knowledge, Himalaya, conservation, high altitude, ethnobotany

## Abstract

**Simple Summary:**

In view of the rising demand for global health care, the traditional medical knowledge is experiencing significant attention to meet the public health needs of developing countries. Knowledge and practice of ethnobotany help to investigate the pharmacological basis of culturally important species of medical value. Traditional knowledge (TK) exists in local indigenous communities and has been passed down over generations as part of community identity. This research attempts to study the plant diversity of medical importance and traditional knowledge interpretation in the Himalayas. This work discloses rich ethnomedicinal knowledge linked with the people residing in the remote location of Northwestern Himalaya. The study justifies the medicinal plants prospective and the inherent knowledge of the local communities. It contains the raw interpretation of 107 high-altitude plants used for disease remedies by taking advantage of the knowledge of the young and old people of the targeted area. The obtained results provide substantial guidance in designating and obtaining the plant material for potential therapeutic interest, as well as sustaining and maintaining the natural ecosystem of the area. It also shows an impetus in discovering the baseline primary data for molecules which would contribute to future drug discovery and disease management, apart from conserving the gene pool of Himalaya and elsewhere in the world.

**Abstract:**

The present study was carried out to enlist the medicinal plants used by the local inhabitants of developing countries such as India, and the district of Kupwara of the Kashmir Himalaya has been targeted. Our research is one of the first study focusing on the statistical evaluation of the cross-cultural analysis between three different communities i.e., Dard, Kashmiri and Gujjar, of the study area. Sampling was carried out in eight villages in 2017 to 2020, and data were collected from 102 informants based on walking transects, to collect plant specimens, and semi-structured interviews. The medical usages of all collected taxa were grouped into 15 disease categories and 81 biomedical ailments. In this study, we documented around 107 plant taxa belonging to 52 families from the local inhabitants of the Kashmir Himalaya, which regulate the livelihood of the people and support cultural ecosystem services. Asteraceae, Rosaceae, Lamiaceae, Malvaceae, Ranunculaceae, Poaceae, Solanaceae, Polygonaceae, Plantaginaceae and Brassicaceae are the top most dominant families. Herbaceous groups of plants were more common than trees and shrubs, and 71.96% of herb taxa were employed as medicine. Liliaceae, Caprifoliaceae and Portulacaceae (FUV = 0.24 each) have the highest family use value (FUV). The most prominent family was Asteraceae (seven genera, nine taxa), followed by Rosaceae and Lamiaceae (six genera, six taxa each). *Persicaria* Mill., *Rheum* L., *Aconitum* L. and *Artemisia* L. were prominent genera. *Valeriana jatamansi* Jones ex Roxb. (47UR), *Fritillaria cirrhosa* D. Don (45UR), *Arisaema jacquemontii* Blume (37UR), *Asparagus racemosus* Willd. (36UR) and *Rumex acetosa* L. (35UR) were the most important plant taxa with reference to use-reports. The ethnomedicinal applications of *Aesculus indica* Wall. ex Cambess., *Solanum pseudocapsicum* L., *Ranunculus hirtellus* Royle and *Cormus domestica* (L.) Spach plant taxa are reported here for the first time from the Himalayan Kashmiri people. We recommend further research on ethnopharmacological application of these newly recorded ethnobotanical plants. The medical usage of the plant was limited to different parts of the plant. In terms of the usage percentage, whole plant (26.17%), leaves (24.30%) and roots (19.63%) were found to have the highest utilization. The powder form (40.19%) was the most frequently employed method of drug/medicine preparation, followed by the utilization of extracted juice and/or other extracts (22.43%). The ICF values range from 0.85 to 1.00. Their use to remedy parasitic problems (PAR) and insect bites (IB) (ICF = 1.0 each) had the maximum consensus mentioned by the informants, although the number of taxa employed under this category was very limited. The different plant taxa used for the treatment of the gastrointestinal problems (GAS) was the most prominent disease category (262 URs, 16.19%, 25 taxa, ICF = 0.90). About 65% of the plant taxa studied is indigenous to the Asia or Himalayan regions, and around 35% is found to be exotic in nature. A strong positive correlation was found between age, gender, educational qualification and medicinal plant knowledge. No significant association was between people of different communities interviewed in terms of medical knowledge of the plants, *p* = 0.347 (>0.05) and χ^2^ = 2.120. No significant difference was found between the number of species documented concerning gender as *p* = 0.347 (>0.05) and χ^2^ =0.885. This study provides the comprehensive status of ethnomedicinal knowledge among three different communities of the study area. This study provided an impetus in discovering the baseline primary data for molecules which would help in drug discovery and management of various diseases, apart from conserving the genepool of plants in the investigated area.

## 1. Introduction

Humans have been using medicinal plants since ancient times and there has been documentation of these uses [1]. First, a qualitative description of plants was carried out, while more recently, the focus has shifted to the analysis of quantitative data by using different indices and using the use-reports of the taxa in question [2]. Traditional medicine is always considered as the total knowledge, human skills and cultural practices of communities based on their beliefs, experiences and theories indigenous to various cultures, employed for the treatment or improvement of both physical and mental well-being [3,4]. The recording and understanding of the historical and botanical links of traditional knowledge (TK), keeping in mind the imminent threat, has received tremendous global attention [5,6]. Some quantitative analysis makes it possible to demonstrate the importance of different characteristics of plants that are the most appreciated within a given society [7]. Developed countries have better access to modern medicines, produced by chemical synthesis, but in many cases, these originate from a molecule of natural origin of a plant or animal. Despite this, the developed world increasingly appreciates the direct use of herbs, combined with modern medical treatments, especially those herbs that have a scientific basis for the cure of minor illnesses [8]. Developing countries still depend heavily on the utilization of medical herbs, and although the traditional knowledge is being lost in many societies [9], the traditional medical systems are very effective in the treatment of several seasonal common ailments [10]. In the last few decades, TK has become fast endangered, and several ethnobiologists are of the opinion that such custodian knowledge may get wiped out forever [11]. The loss of this knowledge can only be stopped with reasoned ethnopharmacological studies that give added value to local popular knowledge to be transferred to the rest of the population, given its clear economic advantages, and manifesting that ethnobotanical knowledge is important throughout the world [12]. Therefore, the dissemination of ethnobotanical knowledge favors the exchange of knowledge between communities and forms a network of culture [1].

Ethnomedicinal knowledge in India is very interesting because of the diverse traditional medical practices [13]. Plant availability and diversity favor the use of these medicinal plants by diverse people independently of their social level, as well as for livestock. The traditional uses of the plants arise from ancient works of literature, such as Charaka Samhita and Ayurveda [14]. The knowledge pertaining the use of medicinal plants gets orally passed to people from one generation to the next generation [15,16,17]. The people of the rural communities have a relatively rich knowledge of the indigenous uses of medicinal plants; however, this knowledge, as well as its associated cultural practices, is gradually decreasing in the new generation [18,19,20]. Therefore, there is an urgent need to investigate the existing practices of indigenous communities, which in the future will provide baseline data for research and can add value to the cultural practices of nomads and other communities, who wholly depend on plants for their livelihoods [12,21]. Ethnopharmacological sciences are related to the detection, assessment, monitoring, and treatment of adverse effects with pharmaceutical products, and are thus important for management programs of genetic resources conservation and land use management plans that might play a crucial role in development [22,23,24]. Ethnobotanical studies have been reported in different parts of India to be very effective and they are necessary to prevent TK from disappearing [25,26,27,28]. Nowadays, the habitat destruction of medicinal plants due to modern agriculture has led to the loss of indigenous knowledge [29,30].

In India, the enormous diversified ethnic groups and a rich repository of biological resources constitute the basis for the greatest market of medical wealth [31,32,33,34]. The forests of India have an enormous diversity of aromatic and medicinal plants [35,36,37]. As much as 18,532 plant taxa have been found in India, and only 7000–7500 of these taxa are utilized by tribal communities in health care [1,38,39,40]. The newly designated union territory Jammu and Kashmir (J&K) has a total area of42,241 km^2^ (available at www.jk.gov.in, accessed on 23 August 2021), is an extension belt of the western Himalayas (India) and shares a border area with Punjab, Himachal Pradesh, China and Pakistan [1,41]. This region is composed of two main provinces, namely, the Jammu province and Kashmir province. The major area of Jammu has a subtropical climate, while the temperate to alpine climate prevails in the Kashmir valleys [42]. The typical elevation gradients range from 300 to 8000 m above mean sea level, and coupled with diverse climate and topography, this makes this region a place ideal for the evolution of different forms of life, including microbes and plants [43,44]. The people of J&K Himalaya residing in the interior regions do not show satisfaction in health care systems. Data records have shown that 53% of respondents are partially satisfied with health care services, 34% of respondents are quite satisfied and 13% of respondents are oblivious to this subject [42]. The Economic Survey Report of 2017 reveals that the region consumes medicines worth approximately $824,997, of which $549,998 is spent in the Kashmir valley alone. Around 63% of the population does not acquire medicine from any source of pharmacy and depends on local natural resources for medicine. The above cited studies have justified very weak medical infrastructures in the study area, which leads to the greater acceptance and belief in folklore medical practices and focused on the utilization herbs and other plant taxa to cure various seasonal ailments [45,46,47,48,49]. In the Kashmir province, a study indicated that 833 plant taxa belonging to 378 genera and 112 families have potential medical properties [50]. Previously published literature provides very little knowledge concerning the ethnobotany of the Kupwara district; nevertheless, few studies have been published from the area. A study carried out by Lone [51] revealed that 48 plant taxa belonging to 30 families are used as a medicine, have cultural value, have a known aroma, and are consumed as wild food. One of the recent findings of Bhat et al. [52] reported 159 plant taxa belonging to 59 families as potent high value ethnomedicinal plants of the Kashmir Himalaya. A previous study conducted in the Langate regions of the district of Kupwara documented 23 high altitude medicinal plants belonging to 23 genera and 19 families and were reported as a rich source of various phytochemical ingredients [53]. A study by Mir [54] suggested 36 plant taxa employed to treat different forms of dermatological disorders, including physical damage and tropical problems such scabies, rash and inflammation.

Scientific papers have recognized that mountainous regions can be hotspots of ethnopharmacologically relevant species diversity [55]. Several papers have also displayed great variation in terms of the main variables associated to the plant cultural importance. Therefore, there is a need to increase the number of case studies focusing on searching for such variables to better understand situations in which each medicinal plants have predictive power over the species’ cultural importance. Considering the specific group of plants for which medicinal uses overlap, much work remains to be performed to discover whether therapeutic or nutritional value is primary when deciding which wild plants to require for medicine or nutraceuticals. For this reason, this study aimed to characterize the traditional applications of wild plants that are important for medicine by local specialists at high altitude regions of Himalayas. Therefore, this research was conducted for assessing the ethnopharmacological knowledge of local people residing in the interior regions of district Kupwara of Jammu and Kashmir.

To date, the Indian Himalayan region has been studied from various perspectives, including research pertaining to ethnic culture, linguistics, ecological environment and climate change, and a large body of scientific data has been generated to document innumerable traditional medical uses of the plant by ethnic communities in the state [56,57,58,59,60,61,62,63,64,65]. In spite of this, limited reliable data exist regarding wild medicinal plant uses within the remote locations; in other words, TK is still not completely explored. Therefore, through this research, key questions could be answered, such as: (a) Are there any association of the number of species documented with respect to demographic characteristics (age, community, gender and educational qualification? (b) How can indigenous and local plant knowledge help in the survival of tribal communities in harsh environments? (c) What challenges do local people face in their health care system? (d) What is the status of locally available medicinal plants? (e) What plant taxa are consumed by the local communities in the Himalayas as medicine, and how can they be prepared? (f) What human-associated functions do these plant taxa provide? (g) How can researchers go beyond searching for drugs and medicines from nature using plant resources?

The aim of the current work is to contribute to a better understanding of wild ethnomedicinal plant uses—as researchers are already doing in many other parts of the world. The specific objectives of the study were to document the use of wild plant resources, with a focus on medical plants, in the Kashmir Himalayan region and to compare (quantitatively and qualitatively) the possible different uses among previously identified socio-cultural groups living in the region, namely the Dard, Gujjar and Kashmiri. This study has contributed to the discussion of diversity of plant usage in a multinational, multilingual and multi-confessional context. The outcome of this research data will be a prerequisite for prospects and other Research and Development (R&D) activities related to the ethnobotany of plants and for future studies on plants. The most promising hypothesis behind this research was to record the pattern of local utilization of medicinal plants in the study area, link science with society by studying plant-people interaction across different communities (Dard, Kashmiris, Gujjars),validate the data with the scientific findings and side-wise align the inventory data with the predictions of the theoretical frameworks from ecology and ethnobotany perspectives, to provide the baseline primary data for drug discovery, conservation purposes and validation with the scientific findings, and side-wise align the inventory data with the predictions of the theoretical frameworks from ecology and ethnobotany perspectives.

## 2. Materials and Methods

### 2.1. Research Site

**Figure 1:**
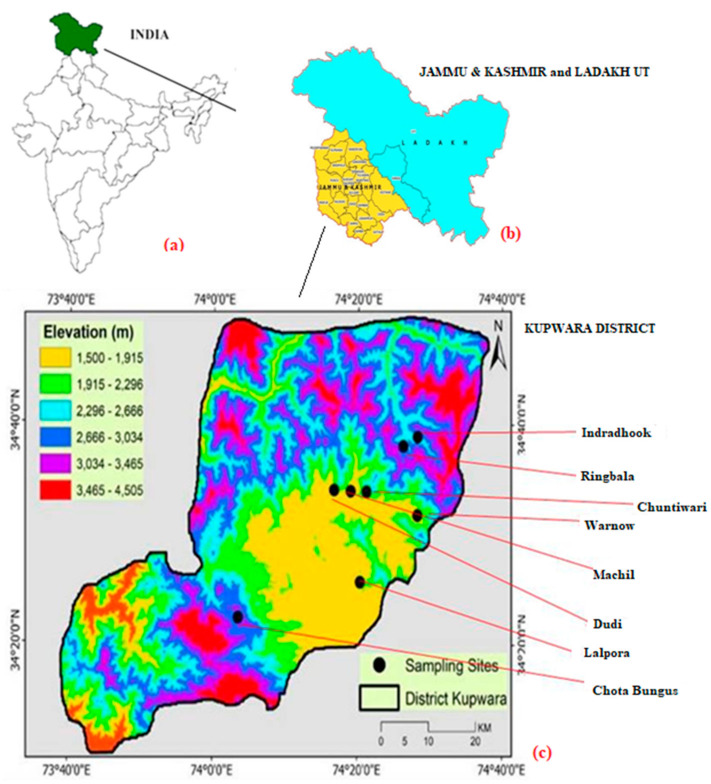
Location map of the study area prepared with the help of ArcGIS (8.1–8.3 Desk top). (**a**) India. (**b**) Jammu & Kashmir and Ladakh ut. (**c**) Kupwara District. (black dots represent the sampling sites with elevations that were visited for data collection).

Kupwara (GPS coordinates: latitude 34°31.5707′ N, longitude 74°15.2768′ E) is a medium-sized district of the Kashmir Himalaya with 870,354 inhabitants and a population density of 368 persons per km^2^ spreads in 3 tehsils and 362 villages (available at https://www.census2011.co.in, accessed on 11 June 2021). This district occupies a total land area of 2379 km^2^ (Figure 1), of which the elevation ranges from 1589 to 4073 m above mean sea level (a.m.s.l.) [66]. The western, northern and eastern belts of Kupwara are hilly, whereas the southern parts are mainly plain. District Baramulla touches, on the east, south and north, the line of control (LOC) of Muzafarabad, Pakistan. The economy of this Himalayan region depends mostly on forest resources and its products. The forest cover is 1464.95 km^2^, which is around 48.76% of the total geographical area [67]. The district is encircled by lofty mountains having thick forests, tough terrains, slopes and snow-clad valleys. The Zojila pass separates the valley of Kishanganga, flowing from east to west, from the main Kashmir Himalaya comprising unique Bungus and Lolab valleys. River Kishanganga and Jhelum serves as the main source of irrigation, for the inhabitants living in this belt of the Himalayas [68]. The district is the home of local Himalayan tribes, such as Dard (occupying the high-altitude regions of Kupwara), Kashmiris (residing in the main valleys and are in majority) and Gujjars (nomad group that surrounds the area and migrates seasonally). The Qazinag range is the main region of IUCN categorized by an endangered wild mammal, the big Markhore goat (*Capra falconeri*) [69]. Paddy, apple and walnut are the main economic crops [70,71]. The area experiences a temperate-cum-Mediterranean climate with the higher regions remaining cold throughout the year; the average temperature varies from −5 to 32 °C [72]. The typical mountainous vegetation varies from subtropical to temperate, alpine to alpine-meadows, interspersed with unique taxa of shrubs and herbs. *Cedrus deodara* (Roxb. ex D.Don) G. Don, *Pinus roxburghii* Sarg., *Abies pindrow* (Royle ex D. Don) Royle, *Morus alba* L., *Platanus orientalis* L., *Picea pungens* Engelm., *Populus alba* L., and *Salix disperma* Roxb. ex D.Don are the main tree components of the western belts of the Himalaya. The dominant shrubby species are *Atropa acuminata* Royle ex Lindl., *Berberis aristata* DC., *Berberis lycium* Royle, *Hyoscyamus niger* L., *Indigofera heterantha* Wall. ex Brandis*, Malva cashemiriana* (Cambess.) Alef., *Parrotiopsis jacquemontiana* (Decne.) Rehder, *Rosa webbiana* Wall. ex Royle and *Sambucus wightiana* Wall. ex Wight & Arn. [73].

### 2.2. Medicinal Plant Survey and Data Collection

#### 2.2.1. Field Expeditions and Recording

**Figure 2:**
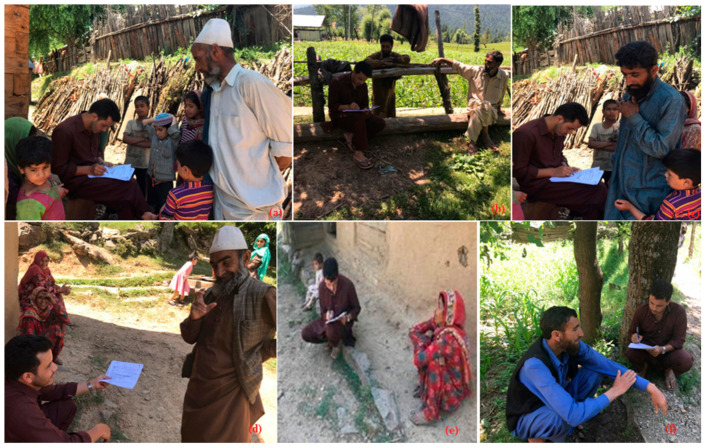
Snapshots during the interviews of local participants for medicinal plants (**a**–**f**).

**Figure 3:**
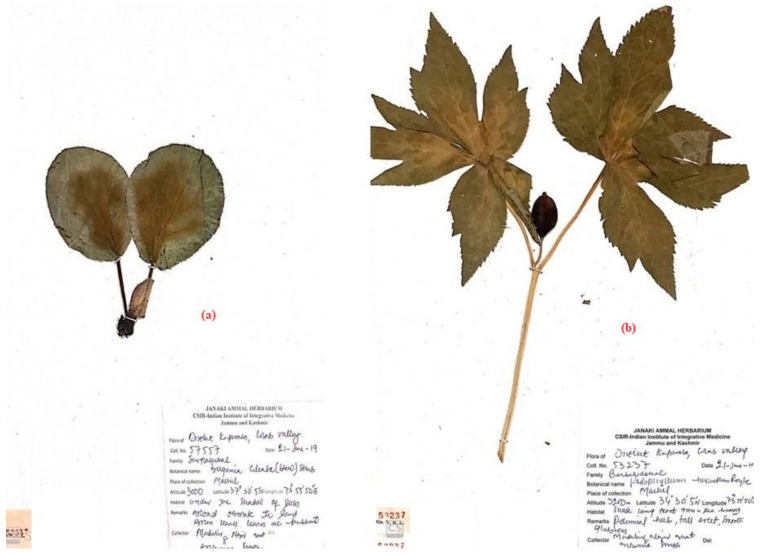
Snapshots of herbarium sheets prepared for a collected voucher specimen, (**a**) *Bergenia ciliata* (Haw.) Sternb., (**b**) *Podophyllum hexandrum* Royle.

From October 2017 to January 2021, field surveys and exploration tours (Figure 2) were conducted for ethnopharmacological documentation covering villages of Chota Bungus, Ringbala, Indradhook, Dudi, Machil, Lalpora, Warnow and Chuntiwari (8) of the district of Kupwara in the Kashmir Himalaya. These villages were selected for this ethnopharmacological study because local people have good knowledge on plants, and they are mostly deprived of medical facilities due to being in extremely remote locations. Demographic data were also extracted from the Census of India 2011 (available at https://censusindia.gov.in, accessed on 22 May 2021). GPS (Manufacturer: Garmin, Country: China) points were collected from the places visited for this study. Plants of medical value were collected in duplicate or triplicate (depending on the availability at specific sites during listing and collecting samples), and herbarium sheets were prepared according to standard protocols [74]; the specimens of the collected medical samples were pressed, dried and pasted on mounting sheets of size 42 × 28 ± 2 cm. Each plants voucher was allotted an accession number from the field diary, specially prepared for the sample collection (Figure 3). A random sampling method was followed [75,76] to collect and investigate sufficient unbiased data samples [77,78,79]. Vouchers specimens were collected and accessioned in the Janaki Ammal Herbarium (acronym RRLH; RRLH stands for Regional Research Laboratory Herbarium) of the CSIR-Indian Institute of Integrative Medicine, Jammu, India. The adopted acronyms for the herbarium code are as per Thiers [80].

#### 2.2.2. Sampling Methods and Informants

Since ethnobotanical investigation was undertaken focusing on the documentation of TK of the local people inhibiting the hilly and the mountainous regions of the Himalaya, we investigated and targeted those inhabitants who practiced self-medication, doctors (herbalist/ayurvedic) and aged headmen of eight selected villages. We also included local field shepherds who have average knowledge of wild plants. Snowball sampling techniques was employed for the selection of informants determining the key persons, and adult family members were selected as participants. The random sampling method of Weckerle et al. [76] was followed to collect sufficient unbiased data coupled with the method suggested by Scheaffer et al. (available at http://www.ru.ac.bd, accessed on 28 May 2021). The efforts were made to investigate the native indigenous inhabitants who knew about the local medicinal plants and forests. A total of 102 local people were interviewed between the ages of 21 to 80 years. There were fewer female (25) than male (77) participants because of the social setup within these communities residing in the interior region of the Kashmir Himalaya (Table 1). The total population recorded during the study period for the eight selected villages was 10,528, and the village Warnow was recorded to be the most populated village, with 2,134 inhabitants, followed by the village Lalpora (2027) and the village Machil (1535). The local language, Kashmiri, was used as the medium of investigation to gather the maximum amount of knowledge for the species in question; additionally, a proper discussion was held to generate more authentic data from the local inhabitants of the study area. Seven resource persons were identified as translators for traditional practitioners, and these people were actively involved in collecting information. The standard questionnaires were formulated based on previous studies and were used for data collection. The regulations by the International Society of Ethnobiology (available at https://www. ethnobiology.net, accessed on 28 May 2021) were followed during the whole study and for data compilation. The information was collected following standard ethnobotanical methods, including free listing, conducting the semi-structured interviews and participatory group discussions with the inhabitants pertaining to the utilization of the plant taxa in question as particular for herbal formulations. Face-to-face interaction for doubtful taxa or taxa that have multiple uses were carried out with the selected informants identified by the resource people. All interviews were conducted in the local language of the communities (Dard, Gujjars and Kashmiris), after which the information were translated into English. Informed consent was obtained verbally from all the informants prior to the semi-structured interviews. Questionnaires prepared for this specific study following standard protocols were applied, and the participants were asked to provide data in questionnaires for the taxa, such as local name, habit, wild/cultivated, parts utilized, ailments (diseases) treated, name of formulations (if any), methods/techniques involved in preparation, side effects (if any) and application period for curing those particular ailments. In the local language, the participants were asked about the local uses of medicinal plants (wild, semi-domesticated and cultivated) in both human and veterinary medicine as well as semi-domesticated and cultivated food plants used in ‘‘unusual’’ ways (i.e., diverging from what those cultivated plants in the Indian Himalayan are typically used for). During the interviews, informants were asked to show the reported medicinal plants (fresh or dried), whenever available. Generated data were utilized for the analysis of the results.

#### 2.2.3. Disease Categorization

The most appropriate way for classifying diseases and remedies based on ethnomedical data largely depends on the research focus, and for most appropriate biomedical screening of the traditional remedies translation of emic to the etic outlook is required [81]. In this study, we followed two approaches, (a) understanding local and indigenous folklore methods, (b) search for unique flora, medical uses and similarities with published data. Keeping in view the application of different approved plant derived drugs and medicine, and the body system used, the specific use categories are formulated. In this study, different diseases that commonly occur in the Himalayan regions were collected from the informants such as doctors, localvaids and hakkims, residing in eight selected villages of the district of Kupwara. All ailments were grouped into fifteen categories, namely, gastrointestinal problems (GAS), cardiovascular problems (CAR), dermatological disorders (DER), cancer (CAN), gynecological problems (GYN), nervous system disorders (GYN), parasitic problems (PAR), respiratory complaints (RES), skeletal-muscular system disorders (SKE), metabolic syndromes (MET), ethnoveterinary ailments (ETH), energy yielding (EY), eyes, ears and nose problems (ENT), fever issues (FVR) and insect bites (IB).

### 2.3. Data Analysis

Six different quantitative ethnobotanical indices, such as species use value, use frequency, family use value, informant consensus factor, relative frequency and relative importance were carried out for analysis of data collected from the field studies. Data were entered into Microsoft Excel© spreadsheets, and then IBM SPSS software version 24.0 (SPSS Inc., Chicago, USA) was used to analyze the data. Statistics were carried out to test the hypothesis. Chi-square tests and proportions were used to assess the association of the number of plant taxa documented concerning the demographic characteristics of the respondents (i.e., age, gender, community and educational qualification). A *p*-value of ≤0.05 was considered statistically significant for all tests, leading to rejection of the null hypothesis, and was validated and taken into consideration.

#### 2.3.1. Species Use Value (UV) and Use Frequency (Fq)

The relative importance of the recorded taxa was calculated through the use value (UV) [82,83]. It was calculated through the following formula:

UV = Σ*U*/*n*

where *U* indicates the number of reports mentioned by each participant for a given plant taxa and *n* is the total number of participants that participated in the study. The UV value varies between 0 for a species that was not mentioned and 1 for a species that was mentioned by all the informants.

To find out the relative importance of various medicinal plant taxa employed for different human-associated disorders, a quantitative index frequency (%) was employed for the use frequency (Fq), which is expressed in a percentage and calculated by multiplying the use-value mentioned by the informants by 100.

#### 2.3.2. Family Use Value (FUV)

FUV helps to identify and signify the use-value of a given medicinal plant family used as a drug or medicine flora particular locality. The family use-value is calculated by employing the formula of Hoffman and Gallaher [84]:

$$\mathrm{FUV}=\frac{\sum \mathrm{UVs}}{\mathrm{ns}}$$

where UV_s_ is the species use value of the plants cited by informants and *ns* represents the total number of plant taxa documented in the family.

#### 2.3.3. Informant Consensus Factor (ICF)

ICF highlights plants of particular cultural relevance and agreement in the utilization of taxa. It helps to identify the variability of the medicinal plants and determine the plant taxa of particular interest. The ailments (diseases) were grouped into categories to analyze ICF, and more ethnopharmacological plant taxa can be identified. ICF was calculated using the formula proposed by Heinrich et al. [85] which is used to test the hypothesis of knowledge homogeny, as follows:

$$\mathrm{ICF}=\frac{\mathrm{Nur}-\mathrm{Nt}}{\mathrm{Nur}-1}$$

where N_ur_ is the number of use-reports (citations) in each ailment category and N_t_ is the number of plant taxa employed for particular ailments. The ICF always ranges from 0 to 1. A high range (nearest to 1) means relatively few taxa are employed by a large number of people, while a low range means participants disagree on the taxa employed within a particular category of ailments [86]. ICF values are always higher when single or few plant taxa are recorded to be used by a maximum number of informants to cure a particular illness.

#### 2.3.4. Relative Importance (RI)

The RI of each medicinal plant taxa was calculated using the number of biological (pharmacology) functions (PH) and the normalized number of body systems (BS). The collected data were structured based on PH attributed to each plant taxa, such as whether the informants suggested plant taxa shows antimicrobial, antiinflammatory, antioxidant, anticancerous, and other functions; specific BS treated includes, for example, fever, rheumatism, asthma, constipation, dysentery, piles, etc. RI was analyzed using the methods proposed by Bennett and Prance [87]:

$$\mathrm{RI}=\frac{\mathrm{Rel}\;\mathrm{PH}+\mathrm{Rel}\;\mathrm{BS}}{2}\times 100$$

where Rel PH represents the relative number of biological functions ascribed for the suggested plant taxa, and Rel BS represents the relative number of body system treated employing a single plant taxon. Rel PH and REl BS can be calculated separately by using the following formulas:

$$\mathrm{Rel}\;\mathrm{PH}=\frac{\mathrm{PH}\;\mathrm{of}\;a\;\mathrm{given}\;\mathrm{plant}\;\mathrm{taxa}}{\mathrm{Maximum}\;\mathrm{PH}\;\mathrm{of}\;\mathrm{all}\;\mathrm{reported}\;\mathrm{plant}\;\mathrm{taxa}}$$

$$\mathrm{Rel}\;\mathrm{BS}=\frac{\mathrm{BS}\;\mathrm{of}\;a\;\mathrm{given}\;\mathrm{plant}}{\mathrm{Maximum}\;\mathrm{BS}\;\mathrm{of}\;\mathrm{all}\;\mathrm{reported}\;\mathrm{plant}\;\mathrm{taxa}}$$

where PH represents the number of documented biological functions for a given taxon, and BS represents the number of body systems treated by a single taxon.

Data collected from the study area were statically analyzed.

### 2.4. Literature Review

#### 2.4.1. Species Identification and Voucher Number

A total of 287 voucher specimens of plant taxa were prepared as an herbarium sample representatives mentioned by the participants as a plant utilized in making herbal formulations were collected during field expeditions and knowledge investigation. The literature, such as scientific journals, book and monographs of ethnomedicinal studies, pertaining to the Himalayas (Kashmir Himalaya, Jammu Division, Cold-Desert Ladakh Himalaya, Uttarakhand and Himachal Himalaya) were used for this study. All collected plant samples, along with photographs, were botanically identified using “Alpine Flora of the Kashmir Himalaya” [88], “Flora of Jammu and Kashmir” [89] and “Ethnobotany of the Himalayas—Kashmir, India” [90]. For a discussion on the bioprospection of medicinal plants in the current scenario with respect to major chemical constituents, as well as biological and pharmaceutical applications, few renowned plant taxa-specific review works were reviewed and presented, wherever applicable. Some popular pharmacopeias, such as The American Herbal Pharmacopeia (www.herbal-ahp.org, accessed on 25 May 2021), British Pharmacopeia (www.pharmacopoeia.com, accessed on 25 May 2021), Encyclopedia on Ayurvedic Medicinal Plants (www.indianmedicinalplants.info, accessed on 25 May 2021) and FRLHT (www.envis. frlht.org, accessed on 25 May 2021), were used in this study. The final list of plants was checked with POWO (www.plantsoftheworld online.org, accessed on 27 May 2021). The identified plant taxa were categorized as fungi, lycophytes and ferns, monocotyledons, dicotyledons and gymnosperms. The families of the plant group are arranged as alphabetically and the species within the family is also arranged alphabetically. For a proper confirmation and authenticity, the Janaki Ammal Herbarium Jammu (acronym RRLH) and the National Botanical Research Institute, Lucknow (acronym LWG) were consulted, such as comparing and consulting the specific herbarium vouchers placed within a particular genus and family, deposited in the herbarium with that of the collected samples. The acronyms of the herbaria are according to Thiers 2020 [80]. Voucher specimens were deposited in RRLH, for future reference study material for researchers and interested scientists. The voucher number of the included plants is mentioned in the present study.

#### 2.4.2. Use of Website Databases

The Google search engine was carried out to get information on worldwide disseminated data, referring databases such as scifinder^®^ (www.cas.org, accessed on 9 August 2021), biological abstract (www.ebsco.com, accessed on 9 August 2021), pubmed (www.ncbi.nlm.nih.gov, accessed on 9 August 2021), worldCat (www.worldcat.org, accessed on 9 August 2021), chemical abstracts (www.guides.lib.utexas.edu, accessed on 9 August 2021), science direct (www.sciencedirect.com, accessed on 9 August 2021), web of science group (www.mjl.clarivate.com, accessed on 9 August 2021) and SciELO (www.scielo.org/en, accessed on 9 August 2021). While fetching data, keywords with respect to botanical name and family, vernacular name and synonyms of the plant taxa were taken into consideration. Author abbreviations of the documented plant taxa reported here are in accordance with the POWO database.

## 3. Results

### 3.1. Characteristics of Participants and Correlation

**Figure 4:**
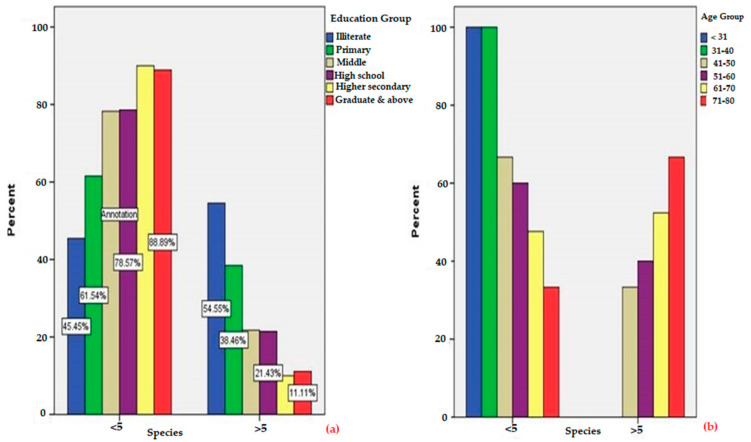
Graphical representation of the relationship between (**a**) education level (χ^2^(5) = 13.734, *p* = 0.017 (<0.05)) and (**b**) age group (χ^2^(5) = 25.673, *p* = 0.001 (<0.05) and knowledge of medicinal plants.

**Figure 5:**
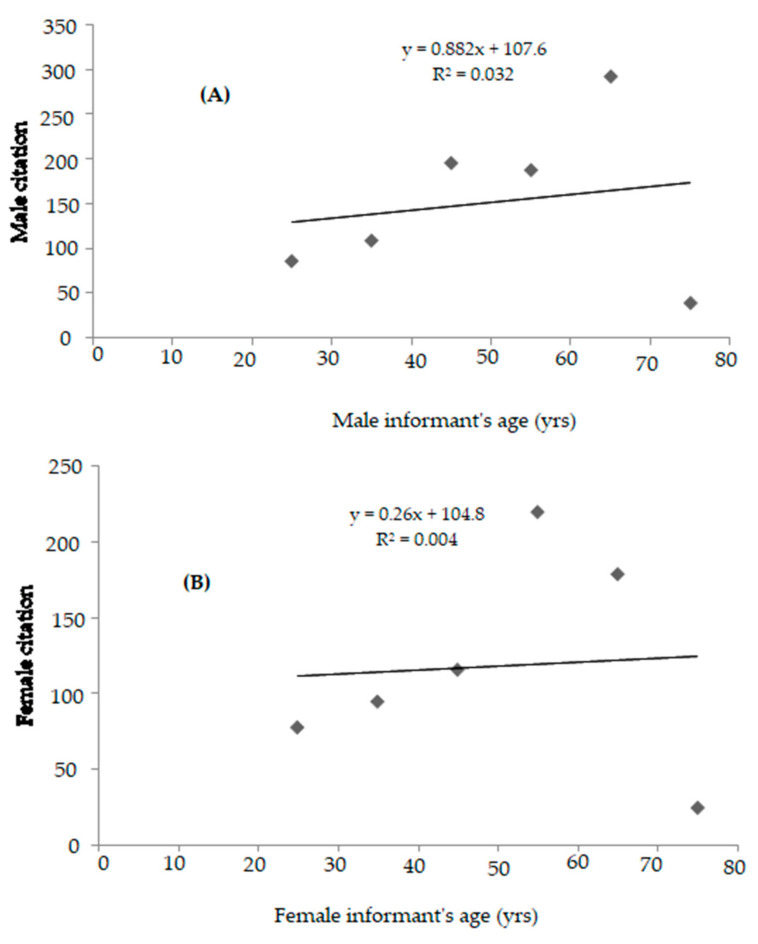
(**A**,**B**) Linear correlation—relative knowledge (use-reports) of informants vs. age of informants; (**A**) male informants, (**B**) female informants.

A total of 102 participants (25 women, 77 men) participated in semi-structured interviews for the current investigation. All selected informants (24.5% women and 75.45% men) were between the age of 21 to 80 years. Based on the educational qualification, it was observed that 24.50% of the informants were illiterate, 17.64% of informants were educated up to the 5th standard, 23.52% of informants were educated up to the middlestandard, 8.82% of informants studied only up to secondary level, 16.67% of informants were educated up to senior secondary level and 8.85% of the informants got their education up to graduate and postgraduate level, reflecting less exposure of people to higher studies due to the social setup in the society and financial barriers. The graphical representation of the relationship between education level (χ^2^(5) =13.734, *p* =0.017, <0.05) (a) and age group (χ^2^(5) =25.673, *p* =0.001, <0.05) (b) and associated knowledge of medicinal plants documented is presented in Figure 4. The detailed association of the number of species documented for demographic characteristics (age, community, gender and educational qualification) is given in Table 2. It was found that there was a significant association between the educational qualification and knowledge of the medicinal plants as χ^2^(5) = 13.734, *p* = 0.017 (<0.05), which shows that educated and highly educated people do not have much knowledge regarding medicinal plants. The reason for this is that these people migrate outside their native places, such as to cities and towns, in search of jobs, employment and for further studies. It has also been observed in the study area that modernizations, including improved and increased road connectivity and improvements of rural infrastructure, has contributed a lot to the decrease of age-old traditional knowledge. It was observed that the knowledge regarding medicinal plant taxa does not show a significant association among different communities as *p* = 0.347 (>0.05) and χ^2^ = 2.120, which shows that the knowledge regarding the medicinal plants is exchanged between the different communities in the study area. No significant difference was found between the number of taxa documented for gender as *p* = 0.347 (>0.05) and χ^2^ = 0.885. It is also interesting to note that in this study, Gujjars reported more medicinal plants compared to Dards and Kashmiris (Table 2), because they prefer plant-based remedies, as they have less access to modern health care facilities, which leads to overharvesting of high-altitude medicinal plants. However, unsustainable use of rare plant species by Gujjar community may be a threat to the conservation of biodiversity. The traditional knowledge of wild edible plants in the Kashmiri community seems to be gradually disappearing because of easy access to modern health care, which may lead to the disappearance of ethnomedicinal knowledge. The association between category of age and medicinal plant’s number documented was also found significant as χ^2^(5) = 25.673, *p* = 0.001(<0.05), making it clear that people with old age are well aware of the uses of medicinal plants growing in the area based on their experience with these native medicinal plants. The relative knowledge (use-reports) of participants versus the age of informants investigated was analyzed as a linear correlation, and is presented in Figure 5A,B. The null hypothesis (Ho2) is rejected (*p*< 0.05) with regards to participants’ knowledge of medicinal plants; however, regression lines for most of the participants have a positive gradient.

### 3.2. Floristic Composition of Medicinal Plants

**Figure 6:**
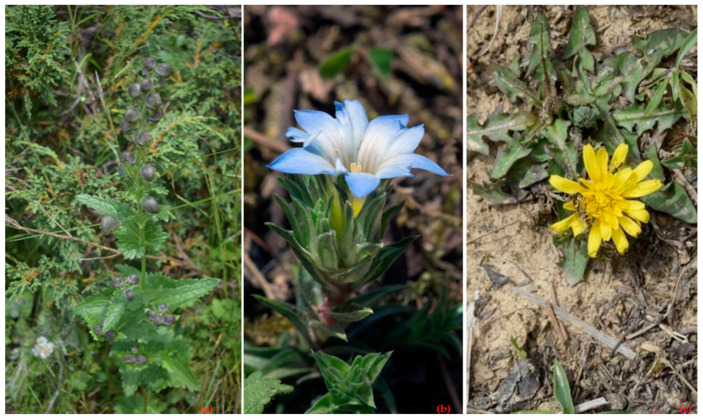
Snapshots of a few of the important high-altitude medicinal plant taxa of the study area (**a**) *Aconitum heterophyllum* Wall. ex Royle, (**b**) *Gentiana argentea* (Royle ex D.Don) Royle ex D.Don, (**c**) *Taraxacum officinale* F.W. Wigg.

In the context of the current survey, numerous wild as well as some cultivated medicinal plants were ethnopharmacologically investigated from the local inhabitants of the Kashmir Himalaya. The investigation indicated that 107 plant taxa from 102 different genera and 52 families are being utilized for medical purposes. These investigated plants were recorded from eight villages of the district of Kupwara, the Kashmir Himalaya as herbal medicine, which are commonly used by the local inhabitants in 15 different categories of diseases. The details of the studied taxa pertaining to botanical names, common name(s), lifeforms, parts of plant utilized, administration techniques, ethnobotanical disease use category, citations mentioned by informants, use frequency (percentage), relative importance and nativity of the taxa employed for ailment treatments are given in Table 3. In terms of habit, 71.96% of the plant taxa employed as herbal medicine is an herb, followed by shrubs and trees (12.15% each), lianas (2.81%) and parasites (0.93%). Out of a total of 107 plant taxa, 82.24% of taxa are categorized as dicotyledons, 11.22% were monocotyledons and 6.5% were non-flowering plants consisting of fungi, gymnosperms, lycophytes and ferns. Most of the plant taxa mentioned by informants were found as wild plants in natural habitat of subtropical, temperate and alpine environment. The most prominent documented families were Asteraceae (seven genera, nine taxa), Rosaceae and Lamiaceae (six genera, six taxa each), Malvaceae and Ranunculaceae (four genera, five taxa each), Poaceae and Solanaceae (four genera, four taxa each), Polygonaceae (three genera, five taxa) and Brassicaceae and Plantaginaceae (four genera, four taxa each), while 42 families were found to be represented by less than two taxa each. The most prominent genera used as medicine were *Persicaria* Mill., *Rheum* L., *Aconitum* L. and *Artemisia* L. Snapshots of some of the important high-value, high-altitude medicinal plants of the study area are shown in Figure 6.

### 3.3. Family Use Value (FUV)

FUV indicated the most biologically significant plant family of any particular region. In the present research, the use value of families represented by more than one plant taxa were calculated and are presented in Table 4. The highest FUV was reported for the families Liliaceae, Caprifoliaceae and Portulacaceae (FUV = 0.24 each), followed by Berberidaceae (FUV = 0.22) and Pinaceae (FUV = 0.21). The least FUV recorded from the study area for the families Malvaceae (FUV = 0.03) and Fabaceae (FUV = 0.07). The family Liliaceae is composed of *Fritillaria cirrhosa* D.Don and *Notholirion thomsonianum* (Royle) Stapf., Caprifolicaeae is represented by *Dipsacus inermis* Wall. and *Valeriana jatamansi* Jones ex Roxb., whereas Portulacaceae includes *Portulaca oleracea* L. and *Lysimachia arvensis* (L.) U.Manns & Anderb. Although the Asteraceae family is represented by the highest number of plant taxa (10) employed for herbal formulations, it has an FUV of 0.17, and the representative taxa under this family were *Arctium lappa* L., *Artemisia absinthium* L., *Artemisia brevifolia* Wall. ex DC., *Artemisia imponens* Pamp., *Cichorium intybus* L., *Dolomiaea macrocephala* DC. ex Royle, *Matricaria chamomilla* L., *Dolomiaea costus* (Falc.) Kasana & A.K.Pandey and *Taraxacum officinale* F.W.Wigg. These taxa were of high value in folklore medicine, and therefore, they were also mentioned by a large number of informants. Acoraceae, Adoxaceae, Apiaceae, Amaranthaceae, Amaryllidaceae, Araceae, Aspleniaceae, Asparagaceae, Dioscoreaceae, Equisetaceae, Campanulaceae, Caryophyllaceae, Convolvulaceae, Crassulaceae, Cucurbitaceae, Cupressaceae, Pteridaceae, Iridaceae, Hammelidaceae, Gentianaceae, Geraniaceae, Juglandaceae, Melanthiaceae, Phytolaccaceae, Morchellaceae, Rhamnaceae, Rubiaceae, Sapindaceae, Saxifragaceae, Scrophulariaceae, Violaceae, Urticaceae and Vitaceae were recorded as monotypic families and are represented by only one plant taxa in the study area.

### 3.4. Plant Taxa, Use-Reports (URs) and Associated Plant Knowledge

In the current study, a total of 107 plant taxa were cited by the informants, and the Machil village (out of eight villages) accounts for the highest relative frequency (Rf = 71.96%), found to have 388 use-reports (URs) (a total of 1631 use-reports were mentioned by informants), representing 77 plant taxa utilized for local drug or medicine preparations. This village was then followed by the Chota Bungus village (Rf = 62.62%, 67 taxa, 361 URs) and Lalpora village (Rf = 52.34%, 56 taxa, 172 URs), which was also based on the proportion of local participants that participated in the interview (Table 1). A total of 90% of the local respondents were of the opinion that all the plants mentioned by them were employed for self-healthcare. The URs in the study area indicate relative significance of herbal medicinal plant taxa for certain categories of uses for curing illnesses. Usually, the highest URs were the most significant, popular and valuable plant taxa employed as medicine by the inhabitants. Based on UVs, the popular plant taxa among the inhabitants of the study area were *Acorus calamus* L., *Ajuga bracteosa*Buch.-Ham. ex D.Don, *Arisaema jacquemontii* Blume, *A. absinthium*, *Asparagus racemosus* Willd., *Bergenia ciliata*(Haw.) Sternb., *C. deodara*, *Codonopsis rotundifolia* Benth., *F. cirrhosa*, *Morchella esculenta* (L.) Pers., *Picrorhiza kurroa*Royle ex Benth., *Podophyllum hexandrum* Royle, *P. oleracea*, *Rumex acetosa* L., *D. costus*, *T. officinale*, *Trachyspermum ammi* (L.) Sprague and *V. jatamansi* (Table 3). Five of the most popular high-altitude taxa, with the highest URs (out of 102 informants), were *V. jatamansi* (47URs), *F. cirrhosa* (45URs), *A. jacquemontii* (37URs), *A. racemosus* (36URs) and *R. acetosa* (35URs). It has been observed that the taxa with the highest URs are most frequently harvested for medical preparation, and therefore, such plant taxa need to be prioritized for conservation and proper management for continuous utilization. Moreover, popular herbal taxa of plants with multiple medical applications and high URs mentioned by users were *A. bracteosa* (32URs), *B. ciliata* (29 URs), *M. esculenta* (29URs), *A. absinthium* (26URs), *A. lappa* (23URs), *Iris kashmiriana* Baker (22URs), *P. kurroa* (27URs), *D. costus* (26URs), *Trillium govanianum* Wall. ex D.Don (21URs) and *B. aristata* (20URs). *Eriolaena candollei* Wall., *N. thomsonianum*, *D. inermis* and *Celtis australis* L. were taxa with very low URs; these taxa were reported to have only 2 URs by the total of 102 informants of the study. The highest use report reflects a higher demand of these medicinal plants in curing various diseases, thus increasing the surplus demand of these medicinal plants, which becomes the main cause of extinction in their natural habitat. *Althaea rosea* L., *M. cashemiriana*, *Malva sylvestris* L. and *M. chamomilla* were recorded with only 3 URs. It has been seen that although these taxa were not popular among the inhabitants, the local medical persons frequently used them in combination with other plants in their local herbal preparations. The medical usages of a few plant taxa, such as *Aesculus indica* Wall. ex Cambess., *Solanum pseudocapsicum* L., *Ranunculus hirtellus* Royle and *Cormus domestica* (L.) Spach, were reported for the first time by the Himalayan Kashmir people, and these will be very useful in future research on medicinal plants.

### 3.5. Plant Parts Used as Medicine

**Figure 7:**
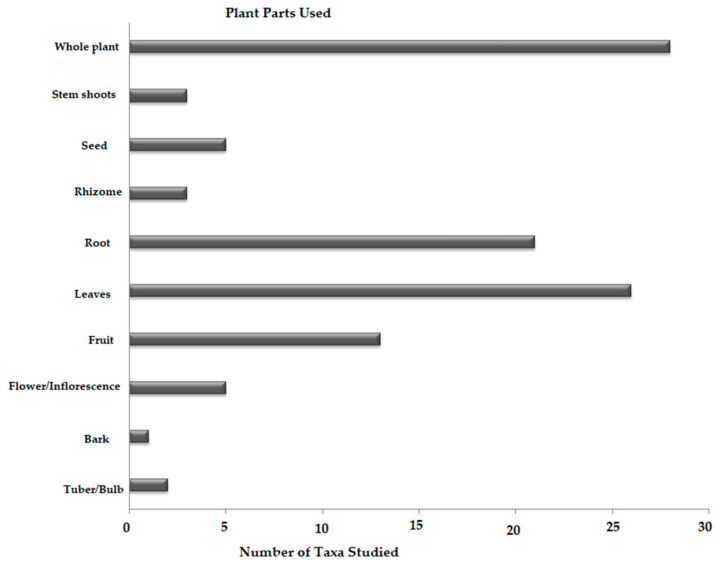
Plant parts used and number of taxa employed as medicine.

The plant taxa diversity of medical administration can be illustrated by their mode of applications. In the study area, various plant parts, such as barks, roots, rhizomes, bulbs, tubers, stems/branches, leaves, seeds, flowers/inflorescence, fruits or even the whole plant, were found to be used by the local inhabitants as a drug/medicine. Most of the plant parts were employed for the treatment of different ailments (illness) and the samples prepared were mostly stored in glass bottles or other containers, as homemade dry powders, which were obtained by crushing down well-dried plant materials; in off-seasons or during heavy snow-falls, they are difficult to obtained from the forests or surrounding areas. Among these, the whole parts of the plants (26.17%) and the leaves (24.30%) are frequently used parts for medicine, followed by roots (19.63%), fruits (12.15%), flowers/inflorescence and seeds (4.67% each) and stems/branches (2.80%). The tubers/bulbs (1.87%) and barks (0.93%) were the least-used parts of the plant for medicine preparation. Figure 7 illustrated the parts of the plant and plant taxa number employed as medicine in the study area.

### 3.6. Methods of Preparation and Administration

**Figure 8:**
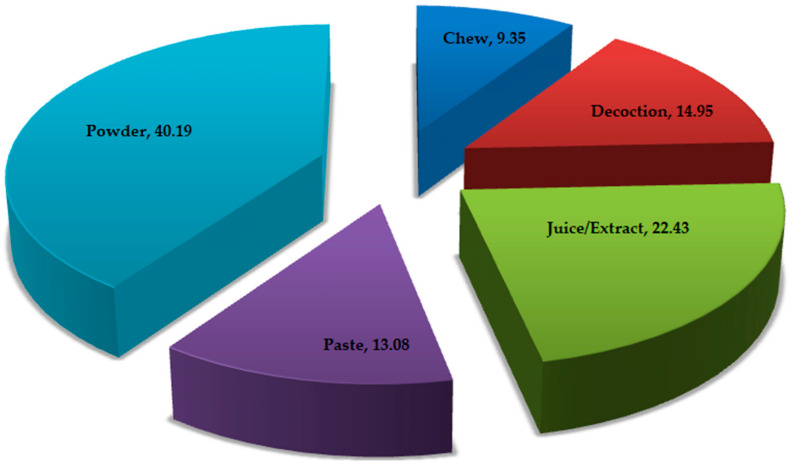
Percentage of different methods of application as a medicine.

The medicinal plant taxa used to treat different illness were prepared by the local inhabitants and administered using various techniques. The utilization of different parts of plants were grouped into five categories (powder, juice/extract, paste, decoction and chew). The use of powder forms was the most preferred method of the used drugs or medicines (40.19%), followed by extracting the juice/extract by crushing fresh materials and mixing them with water (22.43%), use of decoction (14.95%) and by applying paste in the affected regions (13.08%). Using the plants as chewed/raw medicine was documented to be the least common mode of administration from the investigated areas (9.35%) of the Kashmir Himalaya. The extraction of the materials in hot or cold water was the most common method of herbal preparation, and the mode of administration was most commonly oral rather than topical or other forms. In the current study, the herbal practitioners also used some additives besides the plant parts, such as ash, black salt, sugar, honey, onion, zinger, mustard oils, ghee and local home-made wines. Decoctions obtained by boiling the plant materials in water, mostly the roots, rhizomes, barks, etc., were employed in the treatment of internal injuries. For external treatment, pastes or burnt materials along with ghee, honey or mustard oil were used to cure the injuries, and these ingredients also help to reduce the frictions during the application of the remedy. In certain cases, the local healers mentioned that the same parts of medicinal plant taxa can be used for the preparation different ingredients using more than one method. The illustrated figure depicts the percentage of medicinal plant taxa mentioned by key informants employed in different methods of application (Figure 8) for making herbal remedies. In the present study, we found different routes of the administration of herbal preparations. The oral route of administration contributed to about 65% of the total plant taxa (107), which was followed by using rubbing leaves or twigs (18%), taking a bath with boiled water by using plant parts (13%) and the inhalation of smoke (4%).

### 3.7. Informant Consensus Factor (ICF) and Disease Category

**Figure 9:**
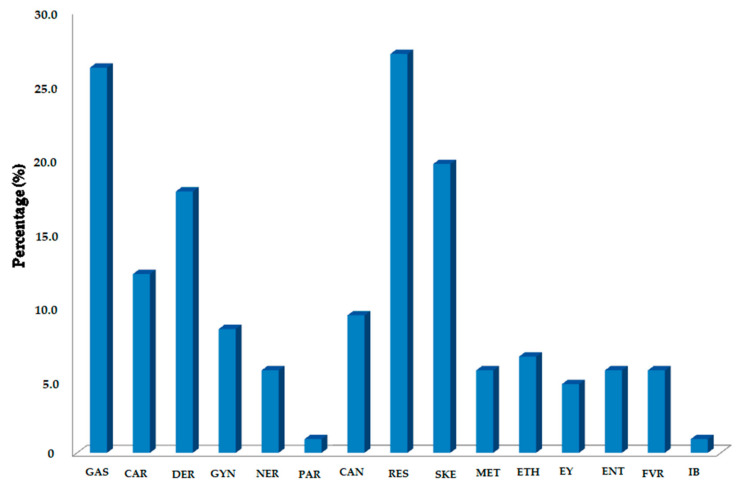
Percentage of plants used in different disease groups. Abbreviation: GAS: Gastrointestinal problems, CAR: Cardiovascular problems, DER: Dermatological disorders, GYN: Gynecological problems, NER: Nervous system disorders, PAR: Parasitic problems, CAN: Cancer, RES: Respiratory complaints, SKE: Skeleto-muscular system disorder, MET: Metabolic syndromes, ETH: Ethnoveterinary ailments, EY: Energy yielding, ENT: Eyes, Ears and Nose problems, FVR: Fever, IB: Insect bites.

The ICF indicates the agreement among participants on the utilization of plants for a particular disease category, and it highlights taxa that have healing potential for specific major purposes. It has been observed that different ailment categories depended on the availability of the plant taxa in the investigated area. In the present study, we have grouped all types of illnesses based on human-system associated disorders into 15 categories, which are presented in Table 5. The results of the ICF values ranging from 0.85 to 1.00, where values near 1 represent a high rate of informant consensus for plant taxa employed against an illness category, whereas an ICF value close to 0 indicates a very low degree of agreement among the respondents for treatment of particular ailment. Basically, ICF can be employed to test the homogeny, or consistency, of a respondent’s knowledge about a particular remedy for a disease category. Parasitic problems (PAR) and insect bites (IB) (ICF = 1.0 each) had the maximum consensus between the informants, although the number of taxa used is only one in both cases. Ethnoveterinary ailments (ETH) (ICF = 0.85) were found to have low consensus agreements between the participants, recorded during the study. As mentioned in Table 5, ICF values for different ailment categories are relatively high, and this indicates that the exchange of knowledge among the participants for the utilization of medical taxa is high, which also proved the apparent efficacy of the recorded taxa. The plants used to treat the gastrointestinal problems (GAS), sub-categorized as antihepatotoxic, antiulcer, choleretic, constipation, diarrhea, abdominal pain, excess gas, heartburn, vomiting and kidney stone, were the most frequently reported (262URs, 16.19%, 25taxa, ICF = 0.90). The most popular plants used for GAS were *P. kurroa*, *Equisetum arvense* L., *Dioscorea deltoidea* Wall. ex Griseb., *T. ammi*, *A. brevifolia*, *Capsella bursa-pastoris* (L.) Medik., *Solena amplexicaulis* (Lam.) Gandhi, *Trigonella foenum-graecum* L., *Abutilon indicum* (L.) Sweet and other plant taxa. This category of ailments was then followed by respiratory complaints (RES), which includes ailments such as asthma, bronchitis, pneumonia, shortness of breath, allergies, tuberculosis and cough (243 URs, 15.02%, 29 taxa, ICF = 0.88). The most popular plants among the inhabitants for RES were *Adiantum capillus-veneris* L., *A. calamus*, *A. jacquemontii*, *Cynodon dactylon* (L.) Pers., *B. aristata*, *Mentha piperita* L., *R. acetosa*, *C. domestica*, *Cydonia oblonga* Mill., *Verbascum thapsus* L., *P. alba*, *Viola odorata* L., *Vitis vinifera* L. and similar other plants in combination with different taxa and ingredients. Other ailment categories with more than 100 use-reports (URs) were dermatological disorders (DER) (203 URs, 19 taxa, ICF = 0.91), skeleto-muscular system disorders (SKE) (175 URs, 21 taxa, ICF = 0.89), cardiovascular problems (CAR) (155 URs, 13 taxa, ICF = 0.92), cancer (CAN) (132 URs, 11 taxa, ICF = 0.93) and gynecological problems (GYN) (101 URs, 9 species, ICF = 0.92) (Figure 9). In this study, it was observed that compared to other disease categories, the informants showed less homogeneity of knowledge with regards to the treatment of gynecological disorders.

### 3.8. Exotic Medicinal Plants

Out of the 107 plant taxa studied, about 65% (69 taxa) of them are indigenous to the Asian or Himalayan regions and 35% (38 taxa) are found to be an alien/invasive in nature. Moreover, these taxa also show affinities with European, Eurasian, African, and American nativity. Some of the documented taxa found to be cultivated by the local inhabitants in their fields and gardens for sustaining their livelihood and to earn their economics. Most popular exotic plants include *Allium sativum* L., *Avena fatua* L., *C. dactylon*, *Oryza sativa* L., *Sorghum halepense* (L.) Pers., *Amaranthus viridis* L., *C. intybus*, *A. lappa*, *T. officinale*, *Nasturtium officinale* W.T. Aiton, *Cannabis sativa* L., *C. australis*, *Stellaria media* (L.) Vill., *Euphorbia royleana* Boiss., *A. indicum*, *Alcea rosea* L., *Ficus carica* L., *M. alba* and various other taxa (Table 3).

### 3.9. Rare Plant Species and Loss of Traditional Knowledge

This study demonstrated that as a result of increasing globalization and modernization, not only erosion of traditional knowledge takes place, it also makes exploration and accessibility easy; this creates a dominant education and research infrastructure, which leads to an increase in the demand of important medicinal plant species, spearheading the discovery of molecules that have pharmacological application. The important medicinal plant species of the area, such as *Aconitum heterophyllum* Wall. ex Royle, *A. chasmanthum*, *Gentiana kurroo* Royle, *P. hexandrum*, *D. costus* and *T. govanianum*, are critically endangered and rare. Overexploitation or overharvesting of these species not only poses a threat to biodiversity, but also a loss of their availability for local traditional medicine.

## 4. Novelty

No other study has been conducted that targets the cross-cultural consensus of three community groups: Dard, Kashmiri and Gujjar in all 10 districts of the Kashmir Himalaya. Moreover, Kupwara is one of the most remote districts of the Kashmir region; the Gujjar and Dard communities reside in high-altitude regions and they have no or little access to any kind of modern medical, infrastructure or transport system. This is in stark contrast with the Kashmiri community, who reside mostly in the plain areas. This disadvantage holds a conservatory influence on traditional knowledge among the Gujjar and Dard communities as compared to people of other communities. In this context, the results presented in this study becomes important for the conservation of traditional knowledge as well as the conservation and management of plant resources. This study also demonstrated the homogenization of traditional knowledge among different communities. The results of a comparative analysis on plants such as *A. indica*, *S. pseudocapsicum*, *R. hirtellus* and *C. domestica* are reported for the first time in the study area. Additionally, the loss of ethnobotanical knowledge is much more pronounced in the younger generation. Therefore, it is of vital importance to rediscover plant sources for food and medicine to improve the overall quality of life for these communities. This study finds that some critically endangered medicinal plants, such as *A. heterophyllum*, *A. chasmanthum*, *G. kurroo*, *P. hexandrum*, *D. costus*, *T. govanianum*, *B. aristata* and *D. macrocephala*, were mostly employed as medicine due to their higher use value in local formulations/herbal preparations. The higher application of these rare and endangered medicinal plants as medicine may cause their overexploitation or elimination, and thus, can indirectly cause traditional knowledge erosion.

## 5. Discussion

The Indian population is approximately 136.64 million, out of which about 40–50% belongs to 550 tribal communities (scheduled castes/scheduled tribes), who speak 325 different languages (available at: statisticstimes.com, (accessed on 24 August 2021)). They mostly reside in remote regions where they lack modern health facilities, and depend on natural resources for their livelihood. Various workers from all over India have contributed their work in the field of medicinal plant conservation and indigenous knowledge. The local hilly inhabitants of the district of Kupwara possess rich knowledge of medicinal plants. In this study, we documented plants with high medical value of 107 different taxa used as drugs or medicine by the local population. In the present findings, we took the advantage of interviewing a wide range of people between 21 to 80 years, to analyze the relationship of age and indigenous knowledge of herbal medicine. In the study area, most of the respondents are of the opinion that traditional healers should always be male, and therefore, 77 respondents in the present study are male, which was supported by a similar study undertaken by Singh et al. [1] in the surrounding area of the Kashmir Himalaya. Several research findings have shown that the ethnomedical knowledge and indigenous cultural practices increase with age, and local inhabitants of the study area above 50 years are the main custodians for cultural practices prevailing in their community [49,53,54]. A strong positive association was found between the education level of the informant and the medical knowledge of the plants (χ^2^ (5) = 13.734, *p* = 0.017 (<0.05)), i.e., highly educationally qualified informants had less knowledge of the traditional systems of medicine, and older people had more knowledge of traditional herbal medicine; this is because the highly educated people become more exposed to modernization [185]. Therefore, we can say that because of increasing modernization, the traditional knowledge is becoming extinct and no longer seems to fit with current reality [186]. Nowadays, health care problems are mainly solved by modern healthcare practices [187]. In this study, it has been observed that the illiterate informants possess more indigenous knowledge compared to educated informants, and therefore, we found a significant negative linear correlation with respect to the medicinal plant taxa and level of education. This result was supported by different studies carried out in nearby areas of the Himalayas and neighboring countries in similar ecosystems [28,43,44,53,57]. The traditional knowledge between the three different communities was exchanged, leading to the homogenization of plant traditional knowledge. Ecological and social factors may have played an important role in the homogenization or exchange of traditional plant knowledge. Again, we mentioned here that the Kashmiri community show restricted use of medicinal plant species as compared to other communities due to the adaptation to a modern life style (erosion of traditional knowledge). The Gujjar and Dard community reported a large number of medicinal plant species, which is linked to their settlement in high-altitude mountains, which have richer biodiversity. The significant amount of knowledge among Gujjar and Dard community reflects a high dependence of these community groups on the local medicinal flora, which poses serious threats such as unsustainable use/overharvesting of traditional medicinal plants. Critically endangered species, such as *A. heterophyllum, A. chasmanthum, G. kurroo, P. hexandrum, D. costus* and *T. govanianum*, need proper conservation and a judicious way of utilization. Thus, from a conservation point of view, this study shows that policy makers should advocate sustainable uses of plant resources.

The documentation of local flora in combination with ethnobotanical knowledge is very important to identify spatial distribution patterns in the diversity and composition of plants to help in sustaining and maintaining such natural ecosystems and traditional knowledge [28,122,188]. In terms of floristic analysis, this study shows that Asteraceae, Rosaceae, Lamiaceae, Malvaceae, Ranunculaceae, Poaceae, Solanaceae, Polygonaceae, Plantaginaceae and Brassicaceae are the most dominant families used for local herbal formulation and medicine. This is supported by other studies in the same area, where the dominance of these families is attributed to their wider distribution, abundance, predominant herbaceous habit and high rate of germination [1,189,190]. Different floras and recent botanical exploration pertaining to the inventorying of plants in the Kashmir Himalaya and elsewhere [1,168,188,191] also reported in the popular dominant families of hilly and mountainous regions are Asteraceae, Fabaceae (Leguminosae), Lamiaceae, Liliaceae, Polygonaceae, Ranunculaceae, Rosaceae and Solanaceae. The reason for the diversity of these families in hilly regions could be: (1) the taxa in such families had a wider distribution range, and could easily adapt due to their herbaceous habit, (2) climatic conditions favor the survival of seedlings and allow plants to bear seeds, which could easily disseminate to new climate areas, or (3) the invasive nature of the plant taxa under these families. Different pharmacopoeias reported these families to be a rich source of alkaloids and flavonoids, which is needed for growth and as building blocks of the body [1], and allows the inhabitants to utilize different taxa of these families as medicine.

The Asteraceae family is considered as the largest family of angiosperms [192,193] and different members of this family were reported to have rich phytochemicals which could possess important biological functions for human and animal studies, such as antitumor, antibacterial, antifungal, antiinflammatory, antioxidant, etc. The phytochemical studies of Asteraceae members indicated the presence of several groups of bioactives, which includes diterpenoids, flavonoids and polyphenols [30,32,43,194]. Earlier studies in the adjoining areas, such as the Bungus valley, also reveals that around 30 plant taxa belonging to 18 families were found to be used in day-to-day life and used as traditional plant remedies for treating various ailments, with the most popular being Asteraceae, Fabaceae (Leguminosae) and Lamiaceae [116]. At the higher altitude of the Kashmir Himalaya, such as the Lolab valley, a study [190] analyzing local peers (herbal saints) and hakims found 20 plant taxa for curing skin problems, and the Solanaceae and Poaceae families dominated the plant list of the Lolab valley [190].

Lamiaceae members (236 genera composed of 6900–7200 different taxa) reported to have a different aromatic (volatile, essential oils, coupled with terpenoids, phenolics, alkaloids and flavonoids) bearing plant taxa [195,196,197] and almost all of them were found to have applications in the food and pharmaceutical industries for the development of value-added flavor and fragrance products useful for human healthcare [1,198,199,200]. The chemical GC/MS investigation of different species indicated the presence of valuable constituents of monoterpene hydrocarbons, oxygenated monoterpenes, sesquiterpene hydrocarbons, oxygenated sesquiterpenes and diterpenes, phenylpropanoids and several non-terpene derivatives, which includes high value constituents such as citral, eugenol, geraniol, menthol, α-phellandrene, *o*-cymene, limonene, eucalyptol, methyl chavicol, linalool, camphor, chavicol, α-cubebene, α-copaene, β-cubebene, β-caryophyllene, α-humulene, γ-muurolene, germacrene D, β-bisabolene, γ-cadinene, thymol and various other phytoconstituents, and these are employed to make cosmetic products, perfumes, flavoring ingredients and medicines [139,140,141,142,201].

In terms of growth forms, the number of herbaceous taxa was followed by shrubs and trees. The frequency for the use of herbs was high, which could be due to the easy availability from nearby forests, which also indicated the abundance of herb taxa as medicine in the natural ecosystems [1]. The published literature indicating the diversity of plants in the Kashmir Himalaya reported herbs as the main constituents in forest ecosystems, and local inhabitants utilize them for their daily needs [88]. Both annual and perennial herbs contain a very high amount of bioactives and different secondary metabolites, which is found to be more effective in curing seasonal disorders [113,202,203].

In the present study, the different taxa recorded to have the highest use-value were *V. jatamansi*, *F. cirrhosa*, *A. jacquemontii*, *A. racemosus*, *R. acetosa* and *A. calamus*. The high use of these taxa as herbal medicine was supported by several findings [1,57,178], which indicated the importance of these plants in the Himalayas and their utility in different communities of tribal Himalayan populations. The local’s applications of *A. heterophyllum* and *A. absinthium,* in various gastrointestinal disorders, have also found a similar use in curing people affected by urinary infections, diarrhea and inflammation [204]. *A. brevifolia* is also used against gastrointestinal disorders. *A. absinthium* is also used against high blood pressure and gastrointestinal ailments [32], and the major phytochemicals reported from this species are lactones and terpenoids (e.g., trans-thujone, γ-terpinene, 1,4-terpeniol, myrcene, bornyl acetate, cadinene camphene, trans-sabinyl acetate, guaiazulene, chamazulene, camphor and linalool [205]. *B. ciliata* is yet another important ethnomedicinal plant for Himalayan tribal communities, which is used to cure ailments such as gastrointestinal disorders, skin diseases and renal/urinary disorders, such as kidney and bladder stones [206], due to the presence of bioactive bergenin, catechin, arbutin, tannic acid, gallic acid and several other phytochemicals [175]. *C. intybus* is yet another important taxon, which is employed against the treatment of gastrointestinal disorders, asthma and gall stones, and grown in many places for the production of inulin on an industrial scale [207]. This species mostly contains saccharides, methoxycoumarin cichorine, flavonoids and essential oils constituents, such as octane, n-nonadecane, pentadecanone and hexadecane.

The consensus among the informants was known by the ICF, which tells us about the homogeneity or agreement of the knowledge regarding medicinal plants among the informants. The ICF was found to be significant for metabolic syndromes, parasitic (antihelminthic) and nervous system-related disorders, followed by gastrointestinal and dermatological diseases. Among the 15 different groups of ailments, eight categories—namely, diseases associated with the eyes, ears and nose, dermatological disorders (abscess, eczema, acne, itching, ringworm, alopecia, blemishes, leukoderma, dandruff, boils, cuts, burns, scabies, wounds), fever (malaria, high body temperature), cardiovascular problems (blood purifier, heart disorders, heart attacks, strokes), gynecological problems (abortifacient, oxytocic, uterine hemorrhage, menstrual problems, genital tract problems, lactation in women), nervous system disorders (anxiety, psychosis, major depressive, narcolepsy, neuropathic pain, vascular dementia), cancer (persistent cough, loss in weight, fatigue), gastrointestinal problems (hepatotoxic, ulcer, choleretic, constipation/indigestion, dysentery, diarrhea, excess gas, heartburn, nausea, stomach pain, vomiting, kidney stone) and metabolic syndromes (obesity, diabetes, blood pressure, excessive body fat, abnormal cholesterol, jaundice)—were recorded to havethe highest ICF values (≥0.90), and the number of popular plant taxa employed for the treatment of each category was 6, 13, 9, 6, 10, 28 and 6 species, respectively. In this study, the ICF values for gastrointestinal disorders were slightly lower with respect to other ailments prevailing in district Kupwara in the Kashmir Himalaya. There are several other similar reported results concerning the plant taxa used for a given illness, such as to treat gastrointestinal disorders, followed by respiratory and dermatological disorders [85,153,208]. This may be due to a high level of gastrointestinal, skin and respiratory diseases due to poor sanitation and open defecation in the area [209].

Inhabitants tend to use leaves for medicines, as opposed to other plant parts, because of the ease of collection and abundant availability [1,165,210]. Moreover, the inhabitants are of the opinion that the leafy parts of the plant store a high quantity of secondary metabolites and essential oils, and therefore, they believe that leaves are better for nutraceutical medicine, phytotherapy or treatment of various health ailments: that is in accordance with [33,105,194,211]. Other studies have shown that leaves are the most preferred plant parts for the preparation of herbal formulations, and the reason could be that leafy parts can be collected more easily than rhizomes, tubers, roots, flowers or fruits [1,56,212]. Similar to leaves, roots are a rich source of bioactive constituents in comparison to other parts of the plant [213]. Inhabitants of the study area make major herbal preparations in the form of powders and decoctions, which is contrast to the findings of other studies [214,215]. The use of pastes is a contemporary method of herbal preparations reported in some previously published studies [216]. The plants collected from the study area were used in different ways, preferably oral. Herbal preparation taken orally is considered useful for the treatment of various internal disorders, whereas for external illness, contemporary application of the drug is used, which is in agreement with previously published studies [217,218,219,220,221,222].

Some of the medical properties mentioned in the present results can be validated by comparing other scientific studies published based on pharmacological assays [223]. The important medicinal plant, *T. officinale*, which is commonly called dandelion, is helpful for a liver disorder. The leaves of the plant are used in diuretics and are a better digestive stimulant [224]. The major phytochemicals present in different parts of the plants includes carotenoids, flavonoids such as quercetin, chrysoeriol and luteolin-7-glucoside, phenolic acids such as caffeic acid, chlorogenic acid and chicoric acid, polysaccharides (inulin), sesquiterpene lactones (taraxinic acid, ixerin D), sterols (taraxasterol, β-sitosterol, stigmasterol) and triterpenes [225]. Findings of this study on *T. officinale* placed this taxon as a plant for a better digestive stimulant, as well as alleviating asthma symptoms and abdominal pain. The endangered medicinal plant *P. kurroa* is found to be effective in improving appetite, and is used to cure various ailments, such as liver problems, spleen disorder, fever and asthma. The people of the study area use these taxa for the antiinflammatory, antihelminthic, refrigerant and stomachic properties. Phytochemical analysis of *P. kurroa* reveals the presence of active iridoids (picroside I and II), cucurbitacins and phenolic components [226], and these active constituents are responsible for their medicinal value. The common plant found in the area, *Datura stramonium* L., is an important hallucinogenic drug and it is reported to be used for memory-related disorders and various skin-related disorders [178]. Externally, this plant is used for burns and rheumatism [227]. Phytochemical studies indicated the presence of tropane alkaloids, such as hyoscyamine and scopolamine, as well as alanine, glutamate, phenylalanine, tyrosine and many other amino acids reported from seeds [228].

This investigation found that many plant taxa are used as medicine for diarrhea, cough, stomach, and abdominal pain. The highest number of taxa was found in the treatment of gastrointestinal diseases, with informant consensus (ICF = 0.90, 28 taxa), which is in agreement with other ethnobotanical studies [229,230]. This may be due to unhygienic conditions, poor supply of (drinking) water, the consumption of spoiled unhygienic food and the practice of open-air defecation [214,231]. The plant taxa used for the treatment of gastrointestinal disorders that have been reported as a rich source of vitamins and flavonoids, along with other nutraceutical ingredients [157,167,171,232,233,234] are *C. rotundifolia*, *C. govaniana*, *P. hexandrum*, *S. pseudocapsicum*, *F. cirrhosa*, *P. hydropiper*, *A. heterophyllum*, *B. aristata*, *L. album*, *A. obtusilobum* and *R. emodi*. These species have anticancer, gastrointestinal, respiratory and dermatological properties. *A. heterophyllum*, *A. absinthium, D. costus* and *B. ciliata* are the taxa that are mainly involved in the treatment of various gastrointestinal diseases [235,236]. *A. heterophyllum* is an important medicinal plant employed for the treatment of ailments related to the nervous system, rheumatism and digestive system [237]. The presence of diterpene alkaloids and flavonoids in the roots of *A. heterophyllum* makes it effective in treating various gastrointestinal diseases, such as diseases of the liver [202]. The extract of roots of *A. heterophyllum* is effective in the treatment of gastric ulcers caused due to cold stress [203]. Traditionally, such taxa help in providing relief from asthma, joint pain, sciatica, cough, neural problems and heart disorders in the study area. As reported in different pharmacological experiments both in vitro and in vivo, *D. costus* exhibited antiinflammatory, antiulcer and hepatoprotective properties, supporting the traditional uses of plants for gastrointestinal solutions [113,237]. The aerial parts of *A. absinthium* are used in many gastric herbal preparations and food supplements to treat various gastrointestinal problems [109]. The rhizomes of *B. ciliata* employed in herbal preparations for gastrointestinal ailments, skin diseases, renal, urinary disorders, oral infections and worm infections [238]. The recorded ICF was also good for respiratory diseases (ICF = 0.88, 29 taxa), and this finding is supported by similar research reported in other studies, which mentioned high ICF for respiratory ailments [207,239]. The results can be attributed to certain factors, such as drastic weather change and a high proportion of moisture, cold, germs and spores, which may lead to abnormalities in the respiratory tract [181,240,241,242]. The plants that were employed mainly for the treatment of respiratory ailments were *D. costus*, *C. impatiens* and *C. domestica,* and were supported by the literature [180]. The ethnobotanical studies of *D. costus* highlighted that this taxon employed in respiratory diseases such as asthma and bronchitis [176,243,244,245]. One of the studies enlists the uses of dried roots of *C. impatiens* to cure respiratory diseases such as asthma and cough [114,160].

Skeleto-muscular diseases (ICF = 0.89, 21 taxa) are common in the study area and plants employed to cure muscular and joint problems are *D. costus*, *B. aristata*, *S. alba* and *V. jatamansi*. These muscular and joint diseases might be due to stress and common injuries [114,160,245]. The roots of *D. costus* are ground to powder and mixed with mustard oil to make a paste, which is then used for the treatment of arthritis [169]. In the traditional system of medicine, *B. aristata* is employed for the treatment of rheumatoid arthritis [246,247]. The human diseases related to the dermatological disorders/skin problems (ICF = 0.91, 19 taxa) also represent the highest number of plant taxa, as was found in other studies [33,248,249], followed by gastrointestinal and respiratory disorders, which may be due to the residents’ exposure to UV radiations at the high-altitude mountainous regions, leading to chronic skin infection, diseases and open-air defecation [250].

*D. stramonium* and *C. deodara* grow abundantly in the study area, and are used by the inhabitants to cure dermatological diseases, as also reported in other studies [48,251]. The fresh extract of the leaves of *D. stramonium* is used on the infected area of the skin to cure wounds, cuts and boils [252]. Deodar extract (volatile oil) distilled from *C. deodara* is mixed with water and/or mustard oil and applied on skin to cure fungal infections [253]. Seeds of ajwain (*T. ammi*) yield essential oil which is employed in the pharmaceutical sector as anantimicrobial, antiinflammatory, antioxidant, cytotoxic, antilithiasis, nematicidal, anthelmintic and antifilarial agent, and has also been reported to have a wide application in the traditional medicine system, where this is used to control bowel disorders such as indigestion, flatulence, colic and diarrhea, because of the presence of active oil components, thymol and carvacrol [254]. Rhizomes of *Rheum emodi* D.Don are locally employed in the treatment of stomachache, fever and cardiac problems. The free anthraquinones, such as physcion, and chrysophanol, rhein, emodin and aloe-emodin were the main active phytochemical constituents of *R. emodi* [104,255]. *C. sativa*, popularly called ‘bhang’ in India, contains cannabidiol and tetrahydrocannabinol (THC), which is utilized as an antipsychotic, schizophrenic and anxiolytic ingredient [256], and this species grows abundantly in the study area. *S. pseudocapsicum* belonging to genus *Solanum*, and so far, 65 species of this have yielded at least 670 compounds [257], and most of the compounds reported have great anticancer and antioxidant activities. Limited data are available on *S. pseudocapsicum*, and this species may have great potential for various pharmacological activities; thus, our study recommends this species for future pharmacological research. There are a number of species of the genus *Ranunculus*, which has been useful traditionally as well as pharmacologically [258]; however, for *R. hirtellus*, as well as other species, not much pharmacological proof currently exists, and thus, this could be a potential plant for future pharmacological research. Moreover, several other studies supported the medical application and bioprospection of the findings on medicinal plants presented in this research and reported to have wide applications in different pharma sectors [106,120].

In the study area, 38 plant taxa (out of 107) were found to be in the invasive category and other results are supported by various recorded studies on alien plants. Most of them are cited as fast-growing invaders in various regions of Indian Himalayas [1]. The literature states that most of the alien taxa have superior phenotypic plasticity compared to the indigenous plants, and thus, they become superior to the native plants in comparison to fitness components, which leads to easy colonization in the disturbed habitat [259,260,261,262,263]. In India, the most popular families of alien taxa include Asteraceae, Poaceae, Brassicaceae, Fabaceae and Lamiaceae [1,264]. Several other published works supported the invasive nature of these families, which includes studies of the invasion pattern of alien species in China by Wu et al. [264], the Kashmir Himalaya in India by Khuroo et al. [265], Randall [266] in Australia, Diez et al. [267] in New Zealand and Lambdon et al., Rosati et al. and Musarella et al. [268,269,270] in Europe. In the present study, the recorded invasive medical taxa usually thrive in human-disturbed habitats, and other studies from different parts of the Himalayas support these findings [271,272,273]. In a study by Khuroo et al. [262], around 8.5% of Indian flora, composed of 1599 plant taxa, are exotic in nature and were categorized as invasive plants; the most popular families were Asteraceae (134 taxa), Papilionaceae (114 taxa) and Poaceae (106 taxa). In the last few decades, the Jammu and Kashmir Himalayas as a whole have been recorded to have a large number of invasive and exotic plant taxa, which may be due to the naturally imposed climate change and biological invasions in natural ecosystems.

## 6. Conclusions

The present survey reveals that the plant knowledge of local people of Kupwara is among the highest in the Kashmir Himalaya. However, this study also reflects the erosion of traditional knowledge among younger generations, which increases the vulnerability of traditional knowledge. With appropriate policies, this loss of precious knowledge can be reduced or recovered. Additionally, the curriculum in schools and creating awareness among children can prevent future erosion of this traditional knowledge, thus encouraging the transmission of knowledge from one generation to another. On the other hand, this study fills the gap of ethnobotanical investigation, and the rich floristic diversity indicates the high potential of traditional knowledge of native people of the area to serve the development of affordable health care. The use frequency and informant consensus factor of the medicinal plants recorded can help in phytochemical and pharmaceutical studies, and can contribute to conservation practices. This research supported the scientific validation of most of the documented taxa for efficient efficacy against the ailment via findings supported by chemical and pharmacological research. This study further confirmed the hypothesis that plants used in traditional medicine are still important in the remote located hills and valleys of the Himalayas. It also supported that the usefulness of a plant taxon in the community culture is directly related to the local abundance, life-forms and seasonal diseases. Scientific standardization pertaining to the dosage, administration and accuracy of disease diagnosis is needed for the validation of traditional knowledge in developing countries. The terrain of Bungus, Lolab and other adjoining valleys of the Kupwara district is very steeps and hilly; local people of this region are still heavily dependent on forest resources for their basic needs. Furthermore, there is a need for legal regulations with regards to conservation and protection. Human activities, such as building houses, dam constructions, over-harvesting and grazing, were detected as the main threats to local biodiversity, and this, together with heavy demand on the market for medical herbs, puts increased pressure on plant taxa of the Himalayas and elsewhere in the world. Therefore, the most threatened and vulnerable plant taxa should be reconsidered and introduced into the study area via legislation or by addressing ecosystem restoration measures. The results mentioned in this research are definitely going to serve as a future reference material for science in the field of systematic, biochemical and pharmacological studies.

## Figures and Tables

**Table 1:** Demographic characteristics of the places studied.

Factor		Study Area (km^2^)								
		Chota Bungus (924)	Ringbala (93)	Indradhook (129)	Dudi (176)	Machil (948)	Lalpora (67)	Warnow (98)	Chuntiwari (75)	Total
GPS Coordinates	Latitude (N)	34°22′20″	34°38′01″	34°33′51″	34°33′53″	34°38′51″	34°25′38″	34°31′43″	34°33′59″	-
	Longitude (E)	74°03′29″	74°27′36″	74°20′31″	74°18′59″	74°27′05″	74°20′21″	74°28′15″	74°16′42″	-
Population *	Male	800	300	300	500	813	1097	1101	731	5642
	Female	684	250	200	467	722	930	1033	600	4886
	Total	1484	550	500	967	1535	2027	2134	1331	10,528
Informants interviewed	Male	5	6	16	8	7	12	14	9	77
	Female	2	2	5	4	1	4	4	3	25
	Total	7	8	21	12	8	16	18	12	102
Education	Graduate	0	1	0	1	0	4	0	0	6
	Illiterate	2	2	9	1	4	2	4	1	25
	Middle	2	2	5	0	1	2	7	5	24
	Post graduate	0	0	0	0	0	3	0	0	3
	Primary	2	2	3	2	2	2	3	2	18
	Matriculation	0	0	1	5	0	1	2	0	9
	Senior Secondary	1	1	3	3	1	2	2	4	17
	Total	7	8	21	12	8	16	18	12	102
Community (Informants)	Dard	7	8	0	12	8	0	0	0	35
	Gujjar	0	0	21	0	0	0	0	0	21
	Kashmiri	0	0	0	0	0	16	18	12	46
	Total	7	8	21	12	8	16	18	12	102
Use-reports mentioned (URs) **		361	174	223	127	388	172	86	94	1631
Plant Taxa Identified ***		67	54	49	34	77	56	29	36	-
Relative frequency (Rf,%)		62.62	50.47	45.79	31.78	71.96	52.34	27.10	33.64	100.00

* Source: Census India 2011 (District Kupwara, Jammu & Kashmir); ** the number of total use-reports cited by participants are1631; *** the number of total plants studied is 107 taxa.

**Table 2:** Chi-square test value table showing the relationship between knowledge of medicinal plants and the demographic characteristics of informants (gender, place, community and age category).

Category		Taxa Reported		Chi-Square Test Value	*p*-Value
		≤5	>5		
Gender	F	15	20	0.885	0.347
	M	54	13		
Place	Chuntiwari	7	13	14.516	0.043
	Dudi	8	12		
	Indradhook	12	21		
	Khurhamma	4	8		
	Lalpora	10	12		
	Machil	6	8		
	Chota Bungus	9	10		
	Warnow	13	18		
Community	Dard	21	12	2.120	0.347
	Kashmiri	19	12		
	Gujjar	9	29		
Education category	Illiterate	15	18	13.734	0.017
	Primary	8	5		
	Middle	18	5		
	Matric	11	3		
	Senior Secondary	9	1		
	Graduate	8	1		
Age category	≤30	22	0	25.673	0.001
	31–40	9	0		
	41–50	12	6		
	51–60	12	8		
	61–70	10	11		
	71–80	4	8		

**Table 3:** Ethnomedicinal plants of the District of Kupwara in the Himalayas, India.

Taxon/Specimen Code	Common Name	Life-Form	Plant Parts Used	Mode of Administration	Ethnobotanicalusage (EU)*	Use-Reports Mentioned By Informants (URs)	Use Frequency (Fq, %)	Relative Importance (RI)	Nativity (N)/Exotic (E)	Earlier Supported Studies
**FUNGI**										
**Morchellaceae**										
*Morchella esculenta* (L.) Pers./RRLH10182	Sedguchchi	H	Wp	Decoction	GAS: 11 CAR: 18	29	28.43	35.23	N	Wagay et al. [91]
**LYCOPHYTES AND FERNS**										
**Aspleniaceae**										
*Asplenium crinicaule* Hance /RRLH24815	Dade	H	Wp	Juice/extract	CAR	16	15.69	38.64	N	Upreti et al. [92]
**Equisetaceae**										
*Equisetum arvense* L./RRLH 24766	Gundum gud	H	Wp	Juice/extract	GAS	12	11.76	87.50	N	Carneiro et al. [93]
**Pteridaceae**										
*Adiantum capillus-veneris* L./RRLH24785	Gawtheer	H	Wp	Powder	RES: 8 GAS: 11	19	18.63	26.14	N	Dehdari and Hajimehdipoor [94]
**MONOCOTYLEDONS**										
**Acoraceae**										
*Acorus calamus* L./RRLH24859	Vai gasnder	H	Rh	Powder	CAR: 10 RES: 18 GAS: 5	33	32.35	39.77	N	Mukherjee et al. [14]
**Amaryllidaceae**										
*Allium sativum* L./RRLH24756	Rohun	H	Bb	Paste	RES: 9 MET: 3	12	11.76	43.18	E	Gupta et al. [95]
**Araceae**										
*Arisaema jacquemontii* Blume/RRLH24773	Noel	H	Tu/Lv	Juice/extract	CAR: 13 DER: 14 RES: 10	37	36.27	57.95	N	Roshan et al. [96]
**Asparagaceae**										
*Asparagus racemosus* Willd./RRLH24869	Satavir	H	Rh	Decoction	GYN	36	35.29	47.73	N	Rahman et al. [97]
**Dioscoreaceae**										
*Dioscorea deltoidea* Wall.ex Griseb./RRLH24782	Kraeth	L	Bb	Powder	GAS: 9 GYN: 4	13	12.75	43.18	N	Dangwal and Chauhan [98]
**Iridaceae**										
*Iris kashmiriana* Baker/RRLH24800	Mazarmund	H	Rh	Powder	SKE: 17 FVR: 5	22	21.57	48.86	N	Amin et al.
**Liliaceae**										[99]
*Fritillaria cirrhosa* D.Don/RRLH25738	Sheeth kar	H	Fr	Chew/Roasted	CAN: 21 DER: 13 GYN: 11	45	44.12	48.86	N	Wang et al. [100]
*Notholirion thomsonianum* (Royle) Stapf./RRLH24321	Shala misri	H	Se	Powder	SKE	2	1.96	26.14	N	Sadiq et al. [101]
**Poaceae**										
*Avena fatua* L./RRLH24887	Kandael	H	Rt	Juice/extract	EY	11	10.78	34.09	E	Khan et al. [20]
*Cynodon dactylon* (L.) Pers./RRLH24863	Dramun	H	Wp	Powder	RES: 9 SKE: 5	14	13.73	35.23	E	Harun et al. [102]
*Oryza sativa* L./RRLH23741	Dhane	H	Fr	Decoction	DER	12	11.76	53.41	E	Cabanting and Perez [103]
*Sorghum halepense* (L.) Pers./ RRLH24858	Durham, garneel	H	Rt	Paste	DER	13	12.75	35.23	E	Khayal [104]
**DICOTYLEDONS**										
**Adoxaceae**										
*Sambucus wightiana* Wall. ex Wight & Arn./RRLH24861	Hapat fal	S	Lv	Paste	SKE: 8 ETH: 4	12	11.76	34.09	N	Amjad et al. [105]
**Amaranthaceae**							0.00			
*Amaranthus viridis* L./RRLH24771	Lessa/Ganhar	H	Lv	Juice/extract	FVR	10	9.80	44.32	E	Ibrahim et al. [106]
**Apiaceae**										
*Trachyspermum ammi* (L.) Sprague/RRLH24784	Ajwain	H	Se	Powder	GAS	24	23.53	35.23	N	Anwar et al. [107]
**Asteraceae**										
*Arctium lappa* L./RRLH24814	Pughood	H	Wp	Juice/extract	CAR: 12 DER: 11	23	22.55	39.77	E	Wang et al. [108]
*Artemisia absinthium* L./RRLH24857	Tethwayen	H	Lv	Decoction	CAN: 8 MET: 11 SKE: 7	26	25.49	48.86	N	Nigam et al. [109]
*Artemisia brevifolia* Wall. ex DC./RRLH24803	Moree	H	Wp	Decoction	GAS	16	15.69	53.41	N	Nigam et al. [109]
*Artemisia imponens* Pamp./RRLH24865	Janglee tethwayeen	H	Wp	Decoction	GAS: 2 PAR: 7	9	8.82	44.32	N	Nigam et al. [109]
*Cichorium intybus* L./RRLH24868	Kasi hand	H	Fr	Chew/Roasted	DER: 7 GYN: 2 ETH: 6	15	14.71	53.41	E	Nwafor et al. [110]
*Dolomiaea macrocephala* DC. ex Royle/RRLH24799	Mera	H	Lv	Powder	GAS: 16 ETH: 5	21	20.59	26.14	N	Negi et al. [111]
*Matricaria chamomilla* L./RRLH24800	Fuk gass	H	Lv	Decoction	RES	3	2.94	48.86	E	Šavikin et al. [112]
*Dolomiaea costus* (Falc.) Kasana & A.K.Pandey/RRLH24765	Kuth	H	Rt	Powder	SKE: 4 GAS: 7 DER: 13 RES: 6	26	25.49	65.91	N	Pandey et al. [113]
*Taraxacum officinale* F.W. Wigg./RRLH24746	Mudaan hand	H	Wp	Paste	DER: 17 EY: 13	30	29.41	62.50	E	Menković et al. [114]
**Berberidaceae**										
*Berberis aristata* DC./RRLH24754	Daenlider	S	St	Powder	RES: 14, SKE: 6	20	19.61	44.32	N	Malik et al. [115]
*Podophyllum hexandrum* Royle/RRLH24770	Wanwangun/kakdi	H	Rt/Lv	Powder	MET: 9 SKE: 4 CAN: 11	24	23.53	53.41	N	Chaurasia et al. [116]
**Boraginaceae**							0.00			
*Arnebia benthamii* (Wall. ex G. Don) I.M. Johnst./RRLH24798	Kahzaban	H	Rt	Chew	CAN: 12, RES: 2	14	13.73	48.86	N	Rana et al. [117]
*Hackelia uncinata*(Royle ex Benth.) C.E.C. Fisch./RRLH20240	Neelan	H	Rt	Chew	DER	12	11.76	35.23	N	Tariq et al. [118]
**Brassicaceae**										
*Capsella bursa-pastoris* (L.) Medik./RRLH24752	Kral mund	H	Wp	Powder	GAS	20	19.61	53.41	E	Hadi et al. [119]
*Cardamine impatiens* L./RRLH19899	Pahal laish	H	Lv	Juice/extract	RES	5	4.90	39.77	N	Rana and Samant [120]
*Nasturtium officinale* W.T.Aiton/RRLH25799	Nag babed	H	Wp	Powder	ETH: 8, SKE: 4	12	11.76	56.82	E	Bullitta et al. [121]
**Campanulaceae**										
*Codonopsis rotundifolia* Benth./RRLH24796	Belphul	H	Wp	Juice/extract	MET	33	32.35	51.14	N	Gupta et al. [122]
**Cannabaceae**							0.00			
*Cannabis sativa* L./RRLH24816	Charas/Bang	S	Inf	Powder	NER	16	15.69	56.82	E	Zou and Kumar [123]
*Celtis australis* L./RRLH24748	Brimij	T	Sh	Juice/extract	CAR	2	1.96	48.86	E	Sufyan et al. [124]
**Caprifoliaceae**										
*Dipsacus inermis* Wall./RRLH24804	Wtkrm	H	Lv	Powder	SKE	2	1.96	26.14	N	Singh et al. [125]
*Valeriana jatamansi* Jones ex Roxb./RRLH26596	Mushkbala	H	Lv	Juice/extract	DER: 31 SKE: 16	47	46.08	56.82	N	Uprety et al. [126]
**Caryophyllaceae**										
*Stellaria media* (L.) Vill./RRLH24808	Nik hak	H	Lv	Powder	CAR	5	4.90	47.73	E	Salam et al. [127]
**Convolvulaceae**										
*Cuscuta cassytoides* Nees ex Engelm./RRLH24856	Kukliporte	P	Wp	Powder	MET: 4 SKE: 9	13	12.75	26.14	N	Gairola et al. [128]
**Crassulaceae**										
*Rhodiola himalensis* (D.Don) S.H.Fu./RRLH20227	Dand jari	H	Wp	Powder	CAN	16	15.69	90.91	N	Zhuang et al. [129]
**Cucurbitaceae**										
*Solena amplexicaulis* (Lam.) Gandhi/RRLH24484	Kharken	L	Rt	Decoction	GAS	8	7.84	38.64	N	Krishnamoorthy and Senguttuvan [130]
**Euphorbiaceae**										
*Euphorbia royleana* Boiss./RRLH24794	Gandi booti	H	Wp	Powder	GAS: 5 SKE: 2	7	6.86	52.27	E	Ashraf et al. [131]
**Fabaceae**										
*Indigofera heterantha* Wall. ex Brandis/RRLH24776	Brand pathri/Zand	H	Se	Paste	RES	4	3.92	26.14	N	Rahman et al. [97,132]
*Trifolium repens* L./RRLH 24793	Pancha/Gulnaksha ka phool	H	Wp	Chew//Roasted	RES	9	8.82	53.41	E	Kolodziejczyk-Czepas [133]
*Trigonella foenum-graecum* L./RRLH29542	Meth	H	Wp	Paste	GAS	7	6.86	39.77	E	Bano et al. [134]
**Gentianaceae**										
*Gentiana kurrao* Royle/RRLH24870	Nelkanth	H	Wp	Paste	FVR	12	11.76	69.32	N	Mubashir et al. [135]
**Geraniaceaeae**										
*Geranium wallichianum* D.Don ex Sweet/RRLH24777	Rathan joti	H	Wp	Powder	ENT: 7 SKE: 9	16	15.69	21.59	N	Qureshi et al. [136]
**Hamamelidaceae**										
*Parrotiopsis jacquemontiana* (Decne.) Rehder/RRLH24819	Poh	S	Rt	Juice/extract	DER	4	3.92	56.82	N	Ali et al. [137]
**Juglandaceae**										
*Juglans regia* L./RLH20859	Dun kul	T	Fr	Juice/extract	SKE	23	22.55	39.77	E	Delaviz et al. [138]
**Lamiaceae**										
*Ajuga bracteosa* Buch.-Ham. ex. D. Don/RRLH24822	Janeadam	H	Lv	Decoction	GAS: 19 ETH: 13	32	31.37	38.64	N	Not found
*Lamium album* L./RRLH24769	Khash khash	H	Lv	Juice/extract	CAR	21	20.59	69.32	N	Kelayeh et al. [139]
*Mentha piperita* L./RRLH24635	Sed guch	H	Lv	Powder	RES	12	11.76	53.41	E	Shah and Mello [140]
*Nepeta cataria* L./RRLH24769	Gansoi	H	Wp	Powder	ETH: 5 MET: 3	8	7.84	52.27	N	Zomorodian et al. [141]
*Prunella vulgaris* L./RRLH24813	Kalyuth	H	Lv	Paste	CAN	8	7.84	30.68	N	Huang et al. [142]
*Thymus linearis* Benth./RRLH24807	Jayind	H	Wp	Powder	GAS: 7 FVR: 5	12	11.76	60.23	N	Qadir et al.[143]
**Malvaceae**										
*Abutilon indicum* (L.) Sweet/RRLH22614	Ronsh pater	S	LV	Powder	GAS	5	4.90	46.59	E	Ravi et al. [144]
*Alcea rosea* L./RRLH22615	Gulkher	S	Inf/Fl	Paste	RES	3	2.94	26.14	E	Fahamiya et al. [145]
*Eriolaena candollei* Wall./RRLH24871	Mushkbala	H	Lv	Paste	GAS	2	0.98	30.68	E	Kamble et al. [146]
*Malva cashemiriana* (Cambess.) Alef./RRLH24783	Sazeposh	S	Fl	Paste	DER	3	2.94	35.23	E	Riyaz et al. [90]
*Malva sylvestris* L./RRLH21484	Sochal	H	Se	Powder	ENT	3	2.94	61.36	N	Hakeem [147]
**Melanthiaceae**										
*Trillium govanianum* Wall. ex D.Don/RRLH25024	Trepater/Surm gunda	H	Rt	Powder	CAN	21	20.59	35.23	N	Rathore et al. [148]
**Moraceae**										
*Ficus carica* L./RRLH24864	Anjeer	T	Lv/Sh/Lt	Juice/extract	GYN: 9 RES: 7	16	15.69	56.82	E	Bouyahya et al. [149]
*Morus alba* L./RRLH24821	Tul	T	Fr/Lv	Chew	GAS	13	12.75	48.86	E	Rodrigues et al. [150]
**Papaveraceae**										
*Corydalis govaniana* Wall./RRLH24797	Bhukesi	H	Wp	Powder	CAN	13	12.75	35.23	N	Sivakumaran et al. [151]
*Papaver somniferum* L./RRLH20326	Khash Khash	H	Sh	Juice/extract	NER	12	11.76	44.32	E	Masihuddin et al. [152]
**Phytolaccaceae**										
*Phytolacca acinosa* Roxb./RRLH24792	Hapat chur	S	Se	Juice/extract	NER	10	9.80	35.23	N	Kumar et al. [153]
**Plantaginaceae**										
*Picrorhiza kurroa* Royle ex Benth./RRLH24867	Kod	H	Rt	Powder	GAS: 16 SKE: 11	27	26.47	26.14	N	Kafle et al. [154]
*Plantago lanceolata* L./RRLH24817	Penkatch	H	Rt	Chew	CAR	12	11.76	44.32	N	Najafian et al. [155]
*Wulfeniopsis amherstiana* (Benth.) D.Y.Hong/RRLH24866	Kakpae	H	Lv	Decoction	DER	11	10.78	48.86	N	Kakar et al. [156]
**Polygonaceae**										
*Persicaria hydropiper* (L.) Delarbre/RRLH24790	Marchawangan gss	H	Rt/Sh	Powder	IB: 6 GAS: 2	8	7.84	21.59	E	Huq et al. [157]
*Persicaria mitis* (Schrank) Assenov/RRLH24818	Chittahola	H	Rt/Sh	Powder	GAS	4	3.92	21.59	E	Cock and Van Vuuren [158]
*Rheum australe* D.Don/RRLH24779	Pamb chalan	H	LV	Paste	CAR	17	16.67	43.18	N	Bhat et al. [52], Kanta et al. [53]
*Rheum emodi* D.Don/RRLH22121	Mamekh	H	Wp	Juice/extract	MET	10	9.80	26.14	N	Pandith et al. [159]
*Rumex acetosa* L./RRLH24778	Chitta ula	H	Lv/Rt	Decoction	RES: 18 EY: 17	35	34.31	44.32	N	Bello et al. [160]
**Portulacaceae**										
*Portulaca oleracea* L./RRLH24375	Nunner	S	Wp	Juice/extract	CAN: 15 CAR: 8 SKE: 10	33	32.35	21.59	N	Zhou et al. [161]
*Lysimachia arvensis* (L.) U.Manns & Anderb./RRLH28948	Janglee aam	H	Fr	Powder	DER: 3 ENT: 9 RES: 3	15	14.71	47.73	E	Al-Snafi [162]
**Ranunculaceae**										
*Aconitum chasmanthum* Stapf ex Holmes/RRLH24795	Mohund	H	Lv/Rt	Powder	GYN	13	12.75	78.41	N	Shyaula [159]
*Aconitum heterophyllum* Wall. ex Royle/RRLH24801	Patrees	H	Rt	Powder	RES: 7 GYN: 11	18	17.65	47.73	N	Shyaula [163]
*Anemonastrum obtusilobum* (D.Don) Mosyakin/RRLH24787	Charisaban	H	Wp	Powder	CAR: 13 NER: 4	17	6.86	55.68	N	Majid et al. [164]
*Aquilegia vulgaris* L./RRLH24862	Kavashud	H	Rt/Inf	Chew	GYN: 2 DER: 9	11	10.78	48.86	N	Malik et al. [165]
*Ranunculus hirtellus* Royle/RRLH21083	Songul	H	Lv	Decoction	ETH: 3 RES: 9	12	11.76	53.41	N	Not found
**Rhamnaceae**										
*Zizyphus mauritiama* Lam./RRLH51077	Brey kul	T	Fr	Decoction	CAR: 21 DER: 3	24	20.59	30.68	E	Akassh et al. [166]
**Rosaceae**										
*Cormus domestica* (L.) Spach/RRLH54217	Chount	T	Fr	Juice/extract	RES	15	14.71	48.86	N	Not found
*Cydonia oblonga* Mill./RRLH21489	Bumb chaent	T	Fr	Juice/extract	RES	12	11.76	44.32	E	Ashraf et al. [167]
*Fragaria vesca* L./RRLH24753	Gonch	H	Fr	Chew	ENT: 13 GAS: 2	15	14.71	44.32	N	Thakur et al. [168]
*Geum elatum* Wall. ex G.Don/RRLH24796	Shonkar	H	Rt	Paste	RES	4	3.92	48.86	N	Chakraborty et al. [169]
*Potentilla indica* (Andrews) Teschem/RRLH21268	Gonch	H	Rt	Powder	RES	11	10.78	47.73	N	Khuda et al. [170]
*Rosa webbiana* Wall. ex Royle/RRLH24780	Janglee gulab	S	Fl	Paste	RES: 8 EY: 4	12	11.76	53.41	N	Alamgeer et al. [171]
**Rubiaceae**										
*Galium aparine* L./RRLH24824	Lothar	H	Wp	Decoction	GAS	9	8.82	26.14	N	Amjad et al. [105]
**Scrophulariaceae**										
*Verbascum thapsus* L./RRLH24755	Hamesh bahar	H	Lv/Rt	Powder	RES	8	7.84	35.23	N	Ali et al. [137]
**Salicaceae**										
*Populus alba* L./RRLH24745	Phrass	T	Lv	Powder	RES	7	6.86	73.86	E	Rashid and Sharma [172]
*Salix alba* L./RRLH24763	Yeer	T	Lv	Decoction	ETH: 2 RES: 6 SKE: 2	10	9.80	21.59	E	Prashith-Kekuda et al. [173]
**Sapindaceae**										
*Aesculus indica* Wall.ex Cambess./RRLH24812	Haandoon	T	Fr	Chew/Roasted	DER: 4 GAS: 5	9	8.82	26.14	E	Not found
**Saxifragaceae**										
*Bergenia ciliata* (Haw.) Sternb./RRLH24775	Zakhmihayat	H	Rt	Powder	ENT: 14 GAS: 11 FVR: 4	29	28.43	39.77	N	Ahmad et al. [174] Bharate et al. [175]
**Solanaceae**										
*Atropa acuminata* Royle ex Lindl./RRLH23347	Brand	S	Wp	Juice/extract	GYN	13	12.75	38.64	N	Hussain et al. [176,177]
*Datura stramonium* L./RRLH24789	Datura	S	Lv	Juice/extract	DER: 7 SKE: 9	16	15.69	44.32	E	Soni et al. [178]
*Hyoscyamus niger* L./RRLH 24772	Hapatagra	S	Fl	Juice/extract	NER	21	20.59	30.68	N	Nadaf et al. [84]
*Solanum pseudocapsicum* L./RRLH21501	Sharbat	H	Fr	Decoction	CAN	7	6.86	30.68	E	Not found
**Urticaceae**										
*Urtica dioica* L./RRLH24762	Soi	H	Rt	Powder	SKE	16	15.69	48.86	N	Dhouibi et al. [179]
**Violaceae**							0.00			
*Viola odorata* L./RRLH24751	Nun posh	H	Wp	Powder	RES: 5 FVR: 18	23	22.55	55.68	N	Mahboubi and Taghizadeh Kashani [180]
**Vitaceae**										
*Vitis vinifera* L./RRLH10183	Dach	L	Fr/Lv	Juice/extract	RES	11	10.78	39.77	E	Arora et al. [181]
**GYMNOSPERMS**										
**Cupressaceae**										
*Juniperus communis* L./RRLH24379	Yathur	T	Rt	Powder	GAS	9	8.82	65.91	N	Bais et al. [182]
**Pinaceae**										
*Cedrus deodara* (Roxb. ex D.Don) G.Don/RRLH24750	Deodar	T	B	Powder	DER: 16 NER: 11	27	26.47	47.73	N	Medinipur and Bengal [183]
*Pinus roxburghii* Sarg./RRLH24860	Chad	T	Rt	Powder	GAS: 4 EY: 11	15	14.71	44.32	N	Kaushik et al. [184]

**Abbreviations used**. **Life-Forms:** T—Tree, S—Shrub, H—Herb, P—Parasite, L—Lianas (climbers/vines). **Plant parts used:** B—bark, Bb—Bulb, Fl—Flower, Lv—Leaves, Fr—Fruits, Se—Seed, Rh—Rhizome, Rt—Root, Sh—Shoot, Inf—Inflorescence, T—Tuber, Wp—Whole plant, GUM—GUM. **ETHNOBOTANICAL USAGE/DISEASE INDICATION**: **GAS—**Gastrointestinal problems, **CAR**—Cardiovascular problems, **DER**—Dermatological disorders, **GYN**—Gynecological problems, **NER**—Nervous system disorders, **PAR**—Parasitic problems, **RES**—Respiratory complaints, **SKE**—Skeletal-muscular system disorders, **MET**—Metabolic syndromes, **ETH**—Ethnoveterinary ailments, **EY**—Energy yielding, **ENT**—Eyes, Ears and Nose problems, **FVR**—Fever issues, **IB**—Insect bites. * Number in front of the use category refers tothe use-reports cited by informants.

**Table 4:** Family use-value (FUV) of the study area.

Family	Taxon	Total Use-Reports (URs)	Use Value (UV)	Family Use Value (FUV)
Liliaceae	*Fritillaria cirrhosa* D.Don, *Notholirion thomsonianum* (Royle) Stapf.	47	0.47	0.24
Poaceae	*Avena fatua* L., *Cynodon dactylon* (L.) Pers., *Oryza sativa* L., *Sorghum halepense* (L.) Pers.	50	0.5	0.13
Asteraceae	*Arctium lappa* L., *Artemisia absinthium* L., *Artemisia brevifolia* Wall. ex DC., *Artemisia imponens* Pamp., *Cichorium intybus* L., *Dolomiaea macrocephala* DC. ex Royle, *Matricaria chamomilla* L., *Dolomiaea costus* (Falc.) Kasana & A.K.Pandey, *Taraxacum officinale* F.W. Wigg.	169	1.66	0.17
Berberidaceae	*Berberis aristata* DC., *Podophyllum hexandrum* Royle	44	0.44	0.22
Boraginaceae	*Arnebia benthamii* (Wall. ex G.Don) I.M. Johnst., *Hackelia uncinata* (Royle ex Benth.) C.E.C. Fisch.	26	0.26	0.13
Brassicaceae	*Capsella bursa-pastoris* (L.) Medik., *Cardamine impatiens* L., *Nasturtium officinale* W.T. Aiton	37	0.37	0.12
Cannabaceae	*Cannabis sativa* L., *Celtis australis* L.	18	0.18	0.09
Caprifoliaceae	*Dipsacus inermis* Wall., *Valeriana jatamansi* Jones ex Roxb.	49	0.48	0.24
Fabaceae	*Indigofera heterantha* Wall. ex Brandis, *Trifolium repens* L., *Trigonella foenum-graecum* L.	20	0.20	0.07
Lamiaceae	*Ajuga bracteosa* Buch.- Ham. ex. D.Don, *Lamium album* L., *Mentha piperita* L., *Nepeta cataria* L., *Prunella vulgaris* L., *Thymus linearis* Benth.	93	0.92	0.15
Malvaceae	*Abutilon indicum* (L.) Sweet, *Althaea rosea* L., *Eriolaena candollei* Wall., *Malva cashemiriana* (Cambess.) Alef., *Malva sylvestris* L.	15	0.15	0.03
Moraceae	*Ficus carica* L., *Morus alba* L.	29	0.29	0.15
Papaveraceae	*Corydalis govaniana* Wall., *Papaver somniferum* L.	25	0.25	0.13
Plantaginaceae	*Picrorhiza kurroa* Royle ex Benth., *Plantago lanceolata* L., *Wulfeniopsis amherstiana* (Benth.) D.Y. Hong	50	0.49	0.16
Polygonaceae	*Persicaria hydropiper* (L.) Delarbre, *Persicaria mitis* (Schrank) Assenov, *Rheum australe* D.Don, *Rheum emodi* D.Don, *Rumex acetosa* L.	74	0.73	0.15
Portulacaceae	*Portulaca oleracea* L., *Lysimachia arvensis* (L.) U. Manns & Anderb.	48	0.47	0.24
Ranunculaceae	*Aconitum chasmanthum* Stapf ex Holmes, *Aconitum heterophyllum* Wall. ex Royle, *Anemonastrum obtusilobum* (D.Don) Mosyakin, *Aquilegia vulgaris* L., *Ranunculus hirtellus* Royle	61	0.61	0.12
Rosaceae	*Cormus domestica* (L.) Spach, *Cydonia oblonga* Mill., *Fragaria vesca* L., *Geum elatum* Wall. ex G.Don, *Potentilla indica* (Andrews) Teschem, *Rosa webbiana* Wall. ex Royle	69	0.69	0.12
Salicaceae	*Populus alba* L., *Salix alba* L.	17	0.17	0.09
Solanaceae	*Atropa acuminata* Royle ex Lindl., *Datura stramonium* L., *Hyoscyamus niger* L., *Solanum pseudocapsicum* L.	57	0.57	0.14
Pinaceae	*Cedrus deodara* (Roxb. ex D.Don) G.Don, *Pinus roxburghii* Sarg.	42	0.41	0.21

**Table 5:** Informant consensus factor (ICF) of common diseases prevalent in the study area.

Disease Category	No. of Taxa (N_t_)	N_t_%	Use-Reports (N_ur)_	N_ur_%	ICF
GAS: Gastrointestinal problems (hepatotoxic, ulcer, choleretic, constipation/indigestion, dysentery, diarrhea, excess gas, heartburn, nausea, stomach pain, vomiting, kidney stone)	28	26.17	262	16.19	0.90
CAR: Cardiovascular problems (blood purifier, heart disorders, heart attacks, strokes)	13	12.15	155	9.58	0.92
DER: Dermatological disorders (abscess, eczema, acne, itching, ringworm, alopecia, blemishes, leukoderma, dandruff, boils, cuts, burns, scabies, wounds)	19	17.76	203	12.55	0.91
GYN: Gynecological problems (abortifacient, oxytocic, uterine hemorrhage, menstrual problem, genital tract problems, lactation in women)	9	8.41	101	6.24	0.92
NER: Nervous system disorders (anxiety, psychosis, major depressive, narcolepsy, neuropathic pain, vascular dementia)	6	5.61	74	4.57	0.93
PAR: Parasitic problems (antihelminthic)	1	0.93	7	0.43	1.00
CAN: Cancer (persistent cough, loss in weight, fatigue)	10	9.35	132	8.16	0.93
RES: Respiratory complaints (asthma, bronchitis, common cold, pneumonia, shortness of breathing, allergies, tuberculosis)	29	27.10	243	15.02	0.88
SKE: Skeleto-muscular system disorder (swelling, arthritis, rheumatic pain, neck pain, back pain, muscle pain, joint inflammation, bone fracture)	21	19.63	175	10.82	0.89
MET: Metabolic syndromes (obesity, diabetes, blood pressure, excessive body fat, abnormal cholesterol, jaundice)	6	5.61	70	4.33	0.93
ETH: Ethnoveterinary ailments (application in treatment of wounds, stomach problems, milk yielding problems, etc., in cows, goats, horses)	7	6.54	40	2.47	0.85
EY: Energy yielding (tonic, laxative)	5	4.67	56	3.46	0.93
ENT: Eyes, ears and nose inflammation problems (throat pain, toothache, sinus infection, bronchitis)	6	5.61	53	3.28	0.90
FVR: Fever (malaria, typhoid, high body temperature)	6	5.61	54	3.34	0.91
IB: Insect bites (bees, scorpion)	1	0.93	6	0.37	1.00

Note: total number of use-reports (citations) is 1631; total number of taxa is 107; N_ur_—number of use-reports (citations); %N_ur_—percentage of use-reports (citations) contributed to the total amount of use-reports by the respective ailment category; ICF—informant consensus factor; N_t_—number of medicinal planttaxa; %N_t_—percentage of the medicinal plant taxa reported for illness category in comparison to the total amount of recorded taxa.

## Data Availability

Not applicable.
